# Frontiers of Membrane Desalination Processes for Brackish Water Treatment: A Review

**DOI:** 10.3390/membranes11040246

**Published:** 2021-03-29

**Authors:** Soraya Honarparvar, Xin Zhang, Tianyu Chen, Ashkan Alborzi, Khurshida Afroz, Danny Reible

**Affiliations:** 1 Department of Chemical Engineering, Texas Tech University, Lubbock, TX 79409, USA; shonar@mit.edu (S.H.); xin1992.zhang@ttu.edu (X.Z.); Tianyu.chen@ttu.edu (T.C.); Khurshida.Afroz@ttu.edu (K.A.); 2 Department of Civil, Environmental and Construction Engineering, Texas Tech University, Lubbock, TX 79409, USA; Ashkan.alborzi@ttu.edu

**Keywords:** brackish water desalination, membrane desalination, reverse osmosis, nanofiltration, electrodialysis, membrane capacitive deionization

## Abstract

Climate change, population growth, and increased industrial activities are exacerbating freshwater scarcity and leading to increased interest in desalination of saline water. Brackish water is an attractive alternative to freshwater due to its low salinity and widespread availability in many water-scarce areas. However, partial or total desalination of brackish water is essential to reach the water quality requirements for a variety of applications. Selection of appropriate technology requires knowledge and understanding of the operational principles, capabilities, and limitations of the available desalination processes. Proper combination of feedwater technology improves the energy efficiency of desalination. In this article, we focus on pressure-driven and electro-driven membrane desalination processes. We review the principles, as well as challenges and recent improvements for reverse osmosis (RO), nanofiltration (NF), electrodialysis (ED), and membrane capacitive deionization (MCDI). RO is the dominant membrane process for large-scale desalination of brackish water with higher salinity, while ED and MCDI are energy-efficient for lower salinity ranges. Selective removal of multivalent components makes NF an excellent option for water softening. Brackish water desalination with membrane processes faces a series of challenges. Membrane fouling and scaling are the common issues associated with these processes, resulting in a reduction in their water recovery and energy efficiency. To overcome such adverse effects, many efforts have been dedicated toward development of pre-treatment steps, surface modification of membranes, use of anti-scalant, and modification of operational conditions. However, the effectiveness of these approaches depends on the fouling propensity of the feed water. In addition to the fouling and scaling, each process may face other challenges depending on their state of development and maturity. This review provides recent advances in the material, architecture, and operation of these processes that can assist in the selection and design of technologies for particular applications. The active research directions to improve the performance of these processes are also identified. The review shows that technologies that are tunable and particularly efficient for partial desalination such as ED and MCDI are increasingly competitive with traditional RO processes. Development of cost-effective ion exchange membranes with high chemical and mechanical stability can further improve the economy of desalination with electro-membrane processes and advance their future applications.

## 1. Introduction

Freshwater supplies are limited and climate change alters their distribution and availability. Meanwhile, the demand for freshwater is continuously growing, exacerbating the pressure on the limited resources. Population growth increases municipal and agricultural consumption and more intensive industrial activities increase freshwater utilization for purposes such as thermoelectric power generation and the extraction of fossil fuels [1,2,3]. A key solution to respond to such growing demands and avoid further deterioration of freshwater supplies is the exploitation of saline water resources. Brackish water is an attractive alternative to freshwater due to its low salinity and abundance in regions facing water scarcity. In the report prepared by US Geological Survey, brackish ground water in the USA is categorized into four main groups, with salinity and dominant ionic constituents as described in Table 1 [4]. Properties of water in none of these groups are suitable for direct use. To reach the water quality requirements for potable and non-potable applications, the organic and mineral content of brackish water should be partially or totally removed. The standards for potable water quality are stricter to avoid health hazards. However, the concern for industrial applications is more related to the potential damage to the equipment and the reduction in productivity due to inorganic and mineral fouling and scaling. A number of existing components in brackish water, e.g., barium and strontium, have very low solubilities that even in trace concentrations may precipitate, resulting in scale formation [5,6]. 

Currently, brackish water is the feed source of 21% of the volume of globally produced desalinated water [7]. Development of energy- and cost-efficient desalination processes helps further expand the use of brackish water. Existing desalination processes are broadly categorized as thermal or membrane techniques [8]. Thermal processes are suitable for seawater desalination and in regions with low fossil fuel prices, such as the Middle East [8]. Membrane processes, however, are preferred for the treatment of low-salinity brackish water [8,9], especially in areas where energy costs are of substantial concern [8,9]. Membrane processes used for brackish water treatment are classified as pressure-driven processes, e.g., reverse osmosis (RO) and nanofiltration (NF), and electro-driven processes, e.g., electrodialysis (ED) and (membrane) capacitive deionization (M)CDI (refers to both CDI and MCDI) [10]. The efficiency of these techniques depends on the salinity and chemistry of the brackish water, size of the plant, and desalination objectives. RO has become the state-of-the-art process for desalination of brackish water at higher salinity in large-scale. However, the energy-efficiency of RO decreases for lower-salinity waters and in small-scale desalination plants [11,12]. NF is capable of selective multivalent ion removal [13,14], encouraging its use for water softening. The selectivity and tunability of ED and (M)CDI provide the opportunity for partial desalination and selective ion removal. These processes are at different stages of development; RO, NF, and ED have been commercialized while (M)CDI is mainly explored at lab-scale. The knowledge and understanding of the capabilities of these processes assist the research community and industrial sectors to further expand the horizons of their applications.

The unique criteria and the challenges associated with each technique should be taken into account when selecting the desalination process for specific applications. Fouling and scaling are the main challenges associated with membrane processes, resulting in reductions in the amount of generated freshwater and an increase in energy consumption. Many studies have focused on improvement of the membrane material, as well as module design and operation, to reduce fouling and scaling. Such efforts may not be able to completely eliminate membrane clogging for feed water with a high fouling and scaling propensity, mandating the use of pre-treatment steps, acid/anti-scalant dosing, and physical/chemical cleaning. A recent review summarized the methods used to assess the fouling and scaling potentials of various feedwaters [15]. These characterization techniques can indicate the likelihood of fouling and evaluate the effectiveness of the proposed mitigation and removal steps. The processes reviewed herein may also face separate challenges depending on their level of maturity. Development of novel membranes with high chemical and mechanical stability as well as superior performance will remain an active research area. Membrane modifications are especially important for advancing electro-membrane processes due to the significant need for reducing fabrication costs and improving the performance of the current ion exchange membranes (IEMs). Improvement of the physical and chemical properties of the electrodes are also vital for further advancing the applications of (M)CDI and ED-based processes. In this article, we review the principles and challenges associated with RO, NF, ED, and (M)CDI and illustrate the capabilities and limitations of each process. We conduct a review of the recent developments and the active research areas for each technology. This review provides a foundation for identifying appropriate fit-for-purpose treatment approaches to reach cost-effective brackish water desalination. 

## 2. Reverse Osmosis

**Figure 1:**
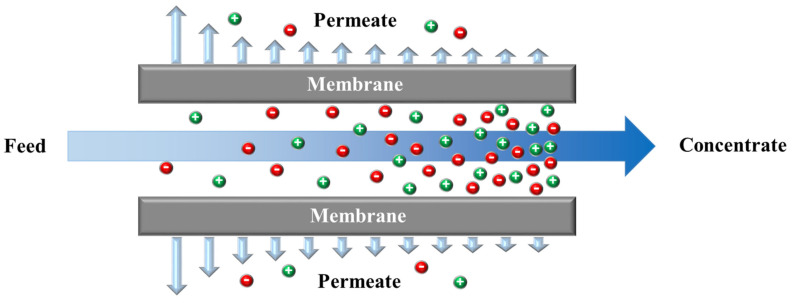
A schematic of a crossflow reverse osmosis (RO) module.

**Figure 2:**
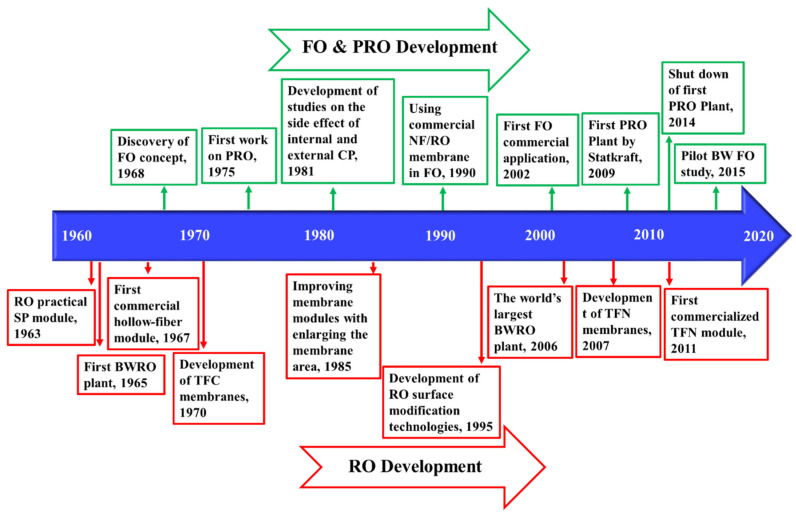
Timeline of the key developments of RO [16,17,18,19,20,21], forward osmosis (FO), and pressure-retarded osmosis (PRO) [22,23,24,25,26,27,28,29,30] processes.

Osmotic pressure gradients between two solutions with different salinity, separated with a semipermeable membrane (that can only pass water), drives water toward the higher salinity. In reverse osmosis (RO), an external pressure is applied to high-salinity feed water, forcing water to flow from the concentrate to the permeate channel as shown in Figure 1. A permeate stream with low salinity and a concentrate stream enriched with salts are the products. RO remains one of the oldest and most utilized approaches for desalination. The first large-scale brackish water RO (BWRO) plant was designed in 1965 [16]. RO and RO-based processes have matured over the past few decades and a summary of their historical developments are provided in the Figure 2. Despite their maturity, the technology has seen efficiency improvements through developments in membranes and process optimization.

### 2.1. RO Membranes

RO membranes exhibit high water flux and more than 99% salt rejection under typical operating conditions [31]. Commercial RO membranes are conventionally synthesized from polyamides (PA) and cellulose acetate (CA) polymers [32]. CA membranes exhibit good salt rejection [33] and chlorine resistance [34] relative to PA membranes, but their sensitivity to pH limits their applications for RO [35]. Thin film composite (TFC) PA membranes contain an ultra-thin cross-linked PA active layer anchored on top of a porous support layer with an interfacial polymerization (IP) reaction. Several authors reviewed the techniques developed for tailoring IP parameters to enrich the properties of PA layers [32,36].

### 2.2. RO Operational Modes and Network Structure

RO is operated in batch, semi-batch or closed-circuit desalination (CCD), and continuous modes. In batch RO (BRO), the concentrate stream flows back to the feed tank. As salt concentrations increase in the concentrate, elevating the osmotic pressure, the applied pressure must also be increased [37]. BRO can provide operational flexibility for operations at a wide range of water recovery [38]. In a CCD process, RO concentrate is mixed with the raw feed water, and fed to the RO membrane. CCD enables high permeate flux, high water recovery, and low pressure. Recycled concentrate increases the salt concentration of the feed; thus, pressure is raised gradually to maintain a constant permeate flux. Once the target production capacity is achieved, or when the required pressure is too high, excess concentrate is directly flushed without recycling, and a new cycle is launched [39]. 

Theoretical studies demonstrate low energy consumption for BRO relative to CCD and single-stage continuous RO [40,41]. Swaminathan et al., [42] demonstrated that energy consumption could be reduced by up to 8% by using a pressurized feed tank in BRO. Moreover, the BRO operation mode allows energy recovery in a single stage without installation of energy recovery devices (ERDs) by feeding pressurized concentrate back into the feed [11]. In CCD, the recirculation of brine results in a decrease in the volume of the concentrate waste [40]. The excess pressure, which is the part of the applied pressure that is above the osmotic pressure of the solution, decreases along the RO module, as opposed to continuous RO that maintains a uniform pressure along the module. In CCD and BRO, excess pressure is time-variant and it continually decreases as the osmotic pressure increases at the outlet. Werber et al., [41] demonstrated that CCD was superior in energy saving compared to continuous RO without using a booster pump and ERD. Lin et al., [43] indicated that CCD consumed more energy than single-stage RO and less energy than multi-stage RO. However, the theoretical and experimental study conducted by Lee et al., [44] showed that incomplete concentrate flushing would increase the specific energy consumption (SEC) of CCD. Higher salinity accumulation in the system can lead to the need for a higher pressure to balance osmotic pressure. Further experimental analysis of Cohen et al., [45] indicated that the CCD saved more energy than continuous RO only under ideal plug flow operation during flushing. They also indicated that CCD had advantages in a wide water recovery range operation by using a single RO system. Such energy consumption analyses determine that batch and semi-batch operations have the potential to improve the energy efficiency of RO for desalination at small-scale and with lower salinity of the feed water, where ERDs lose their efficiency.

RO modules are arranged in serial and parallel configurations. Typically, multi-stage arrays with the membrane modules ratio of 2:1 between two subsequent stages are preferred. Overall, multi-stage design exhibits higher water recovery, while single-stage design has a lower capital cost. RO network configuration determines the cost and efficiency of the process [46]. In general, a serial arrangement of RO modules is preferred due to higher energy efficiency [11]. An inter-stage booster pump is required when a dramatic loss of transmembrane pressure (TMP) is observed, which results in additional energy consumption and capital cost. Recent studies on innovative RO configurations to optimize SEC are mainly focused on BRO and semi-batch RO (CCD) designs.

### 2.3. Energy Recovery Devices in RO

ERDs are used to decrease SEC in RO, especially when using highly efficient devices such as a pressure exchanger (PX). ERDs harvest waste energy from concentrate and significantly reduce the required power for pumping the feed, and hence reduce net energy consumption. Current commercial ERDs consist of a traditional Pelton turbine or reversible pumps and isobaric processes. Traditional reversible pumps and turbines exhibit efficiency losses during energy conversion [12]. Isobaric processes were developed to avoid energy losses during conversion and directly transfer residual pressure of brine to the feed stream. In these processes, a higher-pressure rejected brine pressurizes the lower-pressure bypass of a feed stream by piston positive displacement or rotary direct mixing [12]. Isobaric processes can perform efficiently in wide ranges of flux, temperature, and pressure, while traditional turbine systems are sensitive to process operations out of their design ranges [47]. The application of ERDs for BWRO is less common due to low energy recovery efficiency and low pressure at the feed side. The efficiency of the energy recovery of the current isobaric processes can reach as high as 97%, which promotes the application of ERDs in BWRO. 

### 2.4. Main Challenges of RO

Further development of BWRO faces a series of challenges. The volume of globally produced RO concentrate has exceeded the volume of produced permeate water by ~50% [7]. Thus, economical and environmentally-friendly management of RO concentrate is a significant challenge. Separation efficiency is impacted by membrane properties and fouling conditions. Hence, current research on BWRO is focused on membrane performance improvement, membrane fouling monitoring and prevention, and minimization of the rejected concentrate. 

### 2.5. Approaches to Improve Membrane Performance in RO

RO membrane development is focused on balancing membrane salt rejection and permeate flux. Increasing water flux decreases salt rejection and separation efficiency [48,49]. Chemical and physical modifications of membranes by embedding functional groups are used to enhance water flux, salt rejection, fouling resistance [50], and chlorine tolerance [51,52]. RO membrane surface and substrate functionalization are widely used to improve the separation efficiency. Surface functionalization methods include nanoparticle (NP) doping, functional group grafting, and changing surface morphology to improve membrane performance [21,53]. Various materials have been studied for RO including cellulose [54], aquaporin [55], bentonite [56], carbon-based materials (graphene [57,58], carbon nanotubes (CNT) [59], and carbon quantum dot [60]), bromoacetic groups [46], zeolites [61], metal organic frameworks (MOFs) [62], and metal-based NPs (metals [63,64], metal oxides [65], and metal alkoxides [66]). Grafting hydrophilic groups, e.g., polydopamine [67], polyethylene glycol groups [68,69], and zwitterion groups [70,71], have been reported to improve membrane antifouling properties. Smooth, hydrophilic, and charged surfaces improve antifouling properties and reinforce chlorine resistance [48,72]. Functionalizing the membrane active layer can improve water permeability without significant effects on NaCl rejection [45,47,58], while mitigating biofouling [39,42,44], enhancing membrane anti-bacterial properties [46,63,72], and improving chlorine resistance [73,74].

Substrate layers in membranes act as support for the active layers and provide mechanical strength. Modification of the support layer to improve membrane performance has also been studied by several authors. Lind et al., [61] reported that blending zeolites with the support layer boosted the permeating water flux without significantly impacting salt rejection, due to the sieving mechanism of zeolites. He et al., [75] blended the support layer with amphiphilic copolymer groups to enhance both water flux and salt rejection, and increased the porosity and hydrophilicity of the substrate surfaces. Lee et al., [76] incorporated hydrophilic graphene oxide (GO) groups into the support layer to enhance water permeating flux and membrane fouling resistance. Water permeating flux was optimized by controlling the thickness and the number of hydrophilic groups in the substrate. Kim et al., [77] added silver NPs to substrate layer material and increased bactericidal rates. Though functionalization of active and support layers of membranes successfully improves their performance, the large-scale production of such modified membranes is challenging due to the complex fabrication, high production cost, and limited durability. In addition, the stability and potential release of the incorporated functional groups has not been adequately investigated and may be of environmental concern. 

### 2.6. Approaches to Monitor and Reduce Fouling and Scaling in RO

Membrane fouling and scaling decrease the effective surface area of the membranes and significantly increase their transport resistance. Fouling is classified as organic, inorganic, colloidal, or biofouling. Organic foulants mostly precipitate on the surface of the membrane, blocking pores or forming a cake layer [78]. Inorganic fouling, known as scaling, forms as a result of salt precipitation. Concentration polarization (CP) increases concentration at the membrane–solution interface on the feed side, increasing salt precipitation and scaling potential. Biofouling takes place due to microorganism attachment and growth on the membrane. Furthermore, the co-existence of organic matter and metals exacerbate particle fouling compared to solutions in which only organic matter or metals are present [79,80]. In a solution containing silica and bovine serum albumin (BSA), silica bonded to amino acid groups in BSA and formed a complex that increased fouling [81]. Such synergistic effects necessitate the evaluation of the antifouling performance of membranes for feed water containing a mixture of foulants. 

Pre-treatment steps can be used to reduce membrane clogging in the RO unit. Traditional pre-treatment processes include filtration (membrane filtration and media filtration), softening, inhibitor/anti-scalant addition, coagulation/flocculation, fluid mitigation (flow control, backwash, and air floatation), chemical cleaning (acids, alkalis, detergents, complexing agents, etc.), and disinfection (chlorination, ozonation, ultrasound, and ultraviolet (UV) light) [82,83,84]. Jiang et al., [85] indicated that ultrafiltration (UF)/microfiltration (MF) accounted for 46% of pre-treatment technologies used for RO desalinations, coagulation/flocculation for 18%, disinfection for 13%, and the addition of scale inhibitor for 5%. Many of the traditional pre-treatment methods may cause secondary contamination as a result of the addition of chemicals. Due to their chemical-free nature, electrocoagulation and membrane-based pre-treatment processes have attracted researchers’ attention in recent years.

In electrocoagulation the oxidation reaction at the electrode generates hydrolysis products and hydrogen, leading to the formation of contaminant flocs that are detached from the anodes by the produced hydrogen [86]. Chemical costs for coagulant are avoided in electrocoagulation. However, additional coagulant might be used to facilitate the settling of the particles. Electrocoagulation improves removal efficiency and reduces the amount of sludge generated, compared to traditional coagulation [87]. Electrocoagulation also demonstrated excellent efficiency for removal of silica [88], humic acid (HA) [89], cyanobacteria [90], natural organic matters (NOM) [91], and arsenite [92]. High operational costs and the required maintenance of anodes are the major drawbacks of electrocoagulation.

Forward osmosis (FO) is a pressure-driven membrane process that can be used as a pre-treatment step for RO. In FO, the osmotic pressure gradient spontaneously drives water from low-salinity solution to higher salinity draw solutions. Coupled FO–RO processes can improve the osmotic energy savings from RO brines. Zaviska et al., [93] demonstrated low energy consumption and reduced scaling for a FO–RO hybrid system applied to the desalination of brackish water with high scaling propensity without any other pre-treatment steps. Chun et al., [29] studied a pilot scale FO–RO hybrid system for brackish water desalination with high fouling tendency and found that fouling mitigation strategies were important to maintain the optimal performance of FO, since traditional physical and chemical cleaning were not sufficient to maintain water flux. Fouling remains an important factor related to the performance of hybrid systems at a large scale [94]. 

Pre-treatment steps do not eliminate the scale formation due to the precipitation of CaCO_3_, CaSO_4_, BaSO_4,_ and Mg(OH)_2_ in RO. Scale inhibitors are used to reduce scale formation in industrial desalination processes due to their easy application and relatively low cost compared to other pre-treatment processes [95]. Conventional anti-scalants consist of polyelectrolyte and non-polymeric materials and can effectively inhibit scale formation [96]. The phosphorus and nitrogen content of these anti-scalants can increase concentrate disposal concerns [97] and negatively impact further concentrate management steps, e.g., crystallization [98]. Moreover, deposition of these anti-scalants may increase biofouling on the membrane surface [99]. Therefore, environmentally-friendly and biodegradable “green” inhibitors that are phosphate-free and nitrogen-free have recently gained more attention. Rabizadeh et al., [100] studied the alternative green inhibitors poly(epoxysuccinic acid) and poly(aspartic acid) to control BaSO_4_ scaling. Yu et al., [101] reported the green anti-scalant, carboxymethyl cellulose, to prevent BaSO_4_ scaling. Al-Roomi and Hussain [102] reported organic anhydride-based copolymers to prevent BaSO_4_ scaling in pipes. Hao et al., [103] synthesized carbon quantum dots as green inhibitors to retard scaling of CaSO_4_ and BaSO_4_. Pramanik et al., [104] reported biodegradable non-phosphorus anti-scalant poly(aspartic acid) and its derivatives to control precipitation of CaCO_3_. Optimization of scale inhibitors helps to reduce chemical costs and minimize chemical discharge to the environment [105]. However, a lack of dosing models and economic feasibility evaluation for these green inhibitors has limited their commercial use for the large-scale RO plants, and further research is needed to address these gaps. “Chemical-free technologies” have been proposed to eliminate the addition of anti-scalants. Dayarathne et al., [106] proposed an inhibitor-free method to control CaCO_3_ and CaSO_4_ fouling using micro/nano-sized air bubbles, which minimized CP by increasing mixing and turbulence [107]. Hou et al., [79] used a chemical-free ultrasonic irradiation method to inhibit biofouling. Neither of these approaches have been attempted at a large scale and, in addition, the mechanical and thermal effects of ultrasonic irradiation on membranes are not known.

In situ monitoring of fouling and scaling in RO can significantly help to take appropriate physical/chemical cleaning steps. The real-time monitoring of fouling can be achieved through rheometric and acoustic impedance measurements. In rheometric measurements, the rheological properties at membrane surfaces are measured. Rey et al., [108] monitored CP and membrane fouling using in situ small-angle X-ray scattering, and used in situ micro-particle image velocimetry to measure the rheometric properties at the membrane surface. Meng and Li [109] employed microscopic laser-induced fluorescence to study CP in the system. Concentration distribution was determined by correlating the fluorescent light intensity and fluorescent dye concentration. Ho et al., [110] applied electrical impedance spectroscopy to measure electrical impedance of the fouling layer formed due to colloidal particles in the feed water. Li et al., [111] used ultrasonic time domain reflectometry to describe the deposition of organic matter leading to fouling. The magnitude of the reflected and transmitted ultrasonic waves in these techniques is a function of the acoustic impedance of the media which is related to fouling. The lab-scale monitoring methods have been limited to simple membrane module architecture such as flat-sheet membranes, and the feasibility of these monitoring tools for commercial spiral-wound membrane modules has not been evaluated.

### 2.7. Approaches to Manage RO Concentrate 

A major challenge of BWRO is safe management of the concentrate stream. Conventional BWRO is generally operated at water recovery of up to 85% [112], but depending on feed water quality and operational conditions, the water recovery can be lower. Conventional concentrate management methods include evaporation, surface water discharge, sewer discharge, deep well injection, and crystallization [113]. Concentrate minimization methods were reviewed by Subramani et al., [114] and Giwa et al., [115]. Zero liquid discharge (ZLD) or near-ZLD strategies are designed to minimize concentrate production and maximize water recovery [116,117]. In ZLD desalination, concentrate is treated to recover water or remove salt, and there is no liquid waste discharge to the environment. ZLD is traditionally achieved through energy intensive thermal methods, e.g., evaporation and crystallization. Membrane-based methods are also employed to reach ZLD; however, they often suffer from fouling and scale-up challenges [118]. 

Electro-membrane processes including electrodialysis (ED), electrodialysis reversal (EDR), and electrodialysis metathesis (EDM) are used to increase the overall water recovery and decrease the volume of the concentrate which requires disposal. These processes are described in detail in the subsequent sections. McGovern et al., [119] used a RO–ED hybrid system for brackish water desalination (salinity of approximately 6000 mg/L for RO concentrate) and achieved more than 98% water recovery. Loganathan et al., [120] used an EDR system prior to RO to soften feed water with a salinity of 25,000 mg/L, and reached an overall water recovery of 77%. An evaporator–crystallizer was coupled with an EDR–RO system to manage the remaining concentrate stream. Bond et al., [121] demonstrated low SEC and high water recovery for brackish water desalination with a pilot-scale RO–EDM. The cost of the hybrid system was estimated to be lower than conventional thermal ZLD treatments. In general, ED-based ZLD technologies are promising for RO concentrate management.

Osmosis driven processes, FO, and pressure-retarded osmosis (PRO), can also be used to dewater RO concentrate and, in principle, generate power in return. In PRO, the pressurized, highly saline draw solution is used to drive water from a low-salinity feed stream due to the existence of osmotic gradients [122]. In a typical hybrid RO–FO or RO–PRO process, the rejected brine from RO is used as draw solution for FO and PRO [123]. Altaee et al., [124] simulated an energy- and cost-efficient (NF)–RO–FO hybrid system for the desalination of water with a salinity of about 2400 mg/L, and reached over 90% water recovery. However, the upstream NF process provided 75% of the overall water recovery, while the FO contribution was 15%. Jamil et al., [125] utilized brine with a salinity of approximately 2000 mg/L in five stages of FO to reduce the volume of concentrate by approximately 60%. Lu and Wang [126] used RO brine with a salinity of 12,800 mg/L as the feed solution for FO and reported up to 92.5% water recovery. Lu et al., [127] also coupled FO–RO to treat RO brine and were able to recover 54% water, and the salinity of the brine was approximately 20,000 mg/L. All of these studies, however, were limited to lab-scale. In addition, the long-term tests indicated significant declines in water flux during the period of operation due to fouling. A scaling control strategy is essential for energy-efficient application of FO for dewatering RO concentrate. Anti-fouling FO/PRO membranes are also essential to the performance of hybrid RO–PRO/FO systems. Even though the energy production in FO and PRO partially compensates for the total energy consumption of these systems, the energy efficiency of these processes needs to be improved further [128,129]. Developing self-standing membranes with higher water permeability [130] and better management of fouling are current research directions toward improving RO–PRO/FO performance. In addition, the separation performance of FO and PRO is strongly influenced by the salinity of draw solution. To make the hybrid system economical, the draw solution should be available at a relatively low cost with the possibility of cost-effective regeneration. Chemical constituents of the draw solution should exhibit low toxicity and low viscosity [131]. The cost of the draw solution, energy requirements for its regeneration, and the cost of the disposal of the remaining feed stream of FO/PRO units are significant factors in comparison with other concentrate management techniques.

Membrane distillation (MD) is another alternative approach for concentrating RO brine and increasing the overall water recovery. MD is a thermal process in which the temperature difference between two hot and cold streams increases the vapor pressure gradient, driving water vapor through porous hydrophobic membranes. MD has been coupled with RO to minimize the volume of RO brine and increase water recovery [132,133,134]. However, MD has been mainly studied for managing sea water RO concentrate, with only a couple of studies focusing on BWRO. Martinetti et al., [135] used low temperature MD and FO under similar conditions to recover water from BWRO brines with salinities of 7500 and 17,500 mg/L. The results indicated higher water recovery and a lower fouling tendency for MD–RO compared to FO–RO. The high energy requirements are the primary deterrent to wider use of this approach. Modification of the MD membranes through increasing their porosity or developing a thin hydrophilic layer [136,137] can decrease the conductive heat losses in MD. Dudchenko et al., [138] developed a CNT/polymer composite that could be used as a self-heating membrane in MD that significantly improved its energy efficiency.

Overall, integrating membrane processes to further concentrate RO brine increases the capital cost, overall energy consumption, and operational complexity of the desalination. These approaches can be economical when renewable energy resources are available for powering the desalination processes and also when there is a possibility of salt production and valuable ion recovery. In locations where brine disposal is costly or limited, increasing the water recovery even with higher capital and operational costs becomes inevitable. 

### 2.8. Overall Status of RO for Brackish Water Desalination

Overall, RO is a mature technology suitable for the large-scale treatment of the brackish water with high salinity (above 5000 mg/L). The reported water recovery for existing BWRO plants is relatively low, resulting in relatively large concentrate waste streams. The efficiency of ERDs in RO also depends on the salinity and the volume of the water. With lower salinity feed water and in small-scale desalination, ERDs are less efficient, limiting the potential energy recovery [11,12]. Increasing the water recovery and feed flow rates may further decrease the efficiency of ERDs [11,12]. Even though RO has dominated the existing desalination processes, alternative approaches may have advantages for the treatment of water with lower salinity ranges, desalination for small communities, and selective ion removal. 

## 3. Nanofiltration

**Figure 3:**
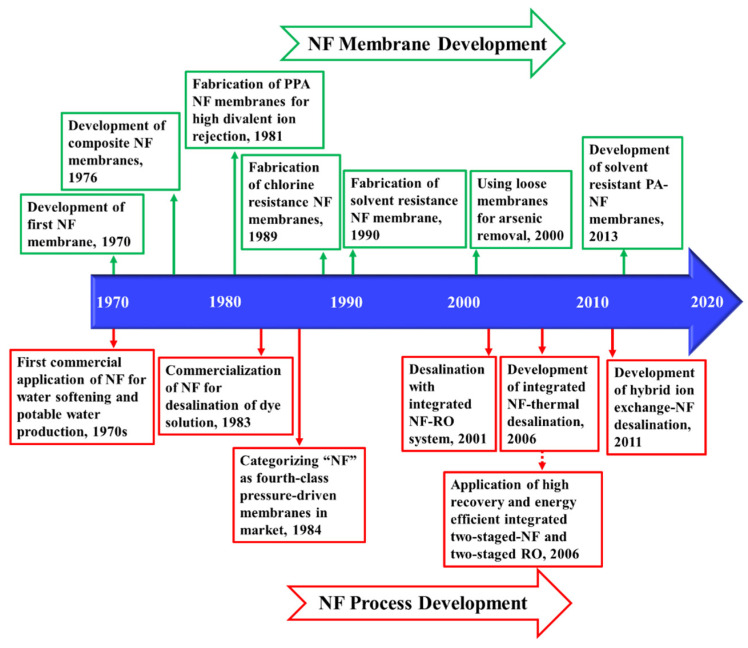
Timeline of the key developments of NF process [142,143,144] and NF membranes [141,145,146].

In nanofiltration (NF), the pressure gradient across the porous membranes is the driving force for separation. NF is operated at 5 to 40 bars, which falls between the operational pressure of UF and RO [139]. The molecular weight cut off (MWCO) which determines the lowest molecular weight of the solute (in Dalton) that a membrane is able to filter is between 200 and 1000 Da, making NF suitable for removing species with a diameter of 0.5 to 2 nm. Hence, NF is capable of rejecting multivalent ions and large organic compounds while passing the majority of monovalent ions [140]. Figure 3 demonstrates the timeline of the key developments of NF from the early commercial application in the 1970s for water softening [141], to the recent development of the hybrid ion exchange-NF desalination process [142]. These developments and research activities are discussed in more detail in the following sections.

### 3.1. NF Membranes

NF membranes are mostly fabricated from PA, polysulfone (PS), polyols, and polyphenols [147]. Recently, inorganic materials such as ceramics, CNTs, and graphene [148,149] have been employed for NF membranes. Although size-related separation is the primary mode of action of NF membranes, Donnan exclusion provides an additional mechanism for hindering co-ion transport through NF membranes with charged surfaces [150]. The approaches employed to improve the properties of NF membranes are summarized in the subsequent sections. 

### 3.2. NF Operational Modes 

**Figure 4:**
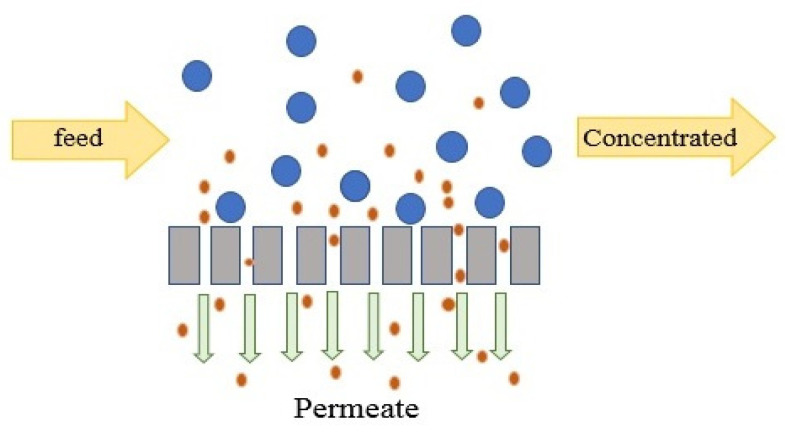
Tangential flow filtration mode.

NF is normally operated in tangential flow filtration (TFF) mode, which minimizes CP in the channel [151]. In TFF mode, feed water flows along the membrane and the permeate stream passes through the membranes. In NF, the membranes are not capable of totally removing all components and particles smaller than 0.5 nm may pass through the membranes to the permeate side, as presented in Figure 4. Hence, as opposed to RO, NF permeate may require further desalination. NF is operated under either constant TMP or constant flux. Constant TMP is a static mode that can be used to evaluate the permeability of clean membranes and estimate the capacity of the unit. Constant flux is a dynamic mode which changes pressure in response to fouling and is mostly used for water with high fouling potential to assure a fixed permeate flux as the TMP increases [152]. The optimum permeate flux is a sustainable flux beyond which fouling rate significantly increases [152]. 

### 3.3. Main Challenges of NF 

Performance of NF is affected by membrane fouling, selectivity, and lifetime. Herein, we review the current research that has been conducted to improve the efficiency of NF. Many of the strategies employed for RO also apply to NF but here, we focus on work specific to NF membranes. 

### 3.4. Approaches to Reduce Fouling and Scaling in NF

Feed water quality and membrane properties such as surface morphology affect fouling and scaling. Membranes with smooth surfaces experience less scaling compared to those with rough surfaces [153]. Combined fouling of NOMs and colloidal particles is greater than their effects separately, although NOMs tend to stabilize the colloidal particles [154]. Similar to RO, physical and chemical cleaning are classical approaches to remove foulants [155,156]. Physical cleanings are generally used for loosely-attached foulants and are divided into three major categories, including hydraulic cleaning, e.g., backwashing and forward flushing [157], pneumatic methods such as air sparging [158], and sonication, in which ultrasound irradiation forces foulants to separate from membranes [159]. Chemical cleaning is also possible, using a variety of chemicals including acids, alkalis, adsorbents, and surfactants, as well as enzymes for biochemical cleaning [160,161]. The effectiveness of membrane chemical cleaning depends on the foulant types and membrane materials [157]. It was shown that the ultrasonic-assisted chemical cleaning of NF membranes significantly improved water recovery for arsenic-rich brackish water treatment [162]. However, the mechanical vibration accompanied by low-pH acidic solution can damage the mechanical and chemical stability of the membranes in the long-term. Here, we review the strategies developed for mitigation of fouling and scaling in NF. We focus on strategies that are unique to NF; however, many of the pre-treatment approaches employed for RO can be applied for NF to reduce fouling and scaling [163,164,165,166].

Self-cleaning and loose membranes can be employed in NF to reduce fouling. Using photocatalytic membranes (PMs), fabricated from the incorporation of photocatalytic materials such as metal-oxides (e.g., TiO_2_ and ZnO) into NF membranes, reduces biofouling due to their superhydrophilicity, high photocatalytic activity, transparency, and electro-conductivity. PMs prevent the adherence of contaminants to the surface of membranes using anti-static forces [167,168]. The dispersion of these catalyst particles in the membrane matrix is challenging, and the improper incorporation of them leads to the reduced separation performance of the membrane. Using loose NF membranes with an average pore size close to 10 nm [169] also reduces the fouling potential. The larger pore size of these membranes, however, reduces the ion removal efficiency, adversely affecting the desalination performance. Such NF membranes are used in dye/salt mixture fractionation and arsenic removal, and also in brackish water treatment for fluoride and NOMs removal [145,170,171].

Surface modification of membranes through the regulation of the physical and chemical properties of the surface is another fouling mitigation approach. The surface properties of membranes are modified through adjusting surface charges (to increase the electrostatic repulsion between foulants and membrane), increasing the hydrophilicity (to decrease the hydrophobic interactions of foulants with the membranes), and reducing the roughness (to create smooth surfaces with minimal contact area for attachment of the fouling components). Van der Bruggen [172] reviewed membrane modifications that have been used with polyether sulfone (PES)-NF membranes, including sulfonation, carboxylation, plasma treating, grafting, and polymer blending to enhance their antifouling properties. Sulfonation and carboxylation of the membranes improves their hydrophilicity through adding sulfonic and carboxylic groups to the polymer substrate. These chemical reagents react with PES via substitution reactions, improving the hydrophilicity of the membrane [173]. Plasma treatment [174] and grafting, e.g., UV, plasma, and ion beam irradiation, enhance the wettability of the membrane surfaces due to the generation of free radicals on the membrane surface, which attracts water. UV irradiation is a straightforward and cost-effective modification method that can be applied to many membranes to increase resistance against organic fouling and biofouling at lab-scale [175]. 

The addition of NPs to the membranes to form thin film nanocomposite (TFN) significantly improves their fouling resistance, as well as their permeability and durability [176,177]. Antifouling nanocomposite membranes containing metal-based NPs, e.g., TiO_2_, Ag, and SiO_2_, and carbon-based NPs, e.g., CNT and GO, have been developed [178]. TiO_2_ and SiO_2_ NPs increase hydroxyl groups and the hydrophilicity of the membrane surface [179,180]. Due to disinfection effects, Ag NPs are commonly used for the biofouling control of membranes, reducing bacterial growth by as much as 90% [181]. Carbon-based nanocomposite membranes have improved anti-fouling, permeability, and desalination capacity [182,183]. Due to the robust pores and hydrophobic interior walls of CNTs, water molecules can easily pass through, leading to high flux and less fouling. Kang et al., [184] demonstrated that embedding sulfonated GO (SGO) into PA-NF membranes improved membrane wettability and fouling resistance. Addition of organic compounds, including polyelectrolytes [185] and coating copolymers, e.g., polyvinyl alcohol (PVA), and poly(oxyethylene) methacrylate (POEM) [186], to the membranes’ surface also improved their antifouling properties due to changes in the roughness. Cyclodextrins [187] and zwitterion polymers [188] have also been used to reduce fouling by making the membranes more hydrophilic, as well as increasing water permeability. However, the performance of TFN membranes depends on many parameters, including membrane material, reaction time, fabrication method, dispersing reagents, and concentration of NPs loaded in the membrane matrix. Particle agglomeration is a major challenge in the fabrication of TFN membrane that can lead to surface defects and consequently reduce the salt removal efficiency of the processes. NP modifications, using proper reagents, and optimizing the fabrication conditions might help with the dispersion of NPs in the polymeric solutions. Optimizing the concentration of embedded NPs is a critical factor, since even though loading a high concentration of NPs may increase the permeation flux of NF membranes, it can reduce salt removal efficiency [189].

### 3.5. Approaches to Enhance Selectivity of NF

The selective removal of organic compounds and multivalent ions in NF make the approach applicable to water softening. Pore size and surface charge of the membranes as well as ion hydration shell can impact compound rejection and membrane selectivity. Ions with weaker hydration shells, e.g., Na^+^, can detach from their hydration shells and pass through NF membranes under operational pressure, whereas those with stronger hydration shells are rejected, e.g., Mg^2+^ [190]. NF typically rejects 95% of divalent ions and 20–80% of monovalent ions. However, the selectivity of NF can further be improved via the approaches described below.

Embedding NPs into the membrane matrix affects membrane pore size and the associated surface thickness/porosity ratio, hence improving ion rejection and selectivity in the process [179,191]. Zareei et al., [192] demonstrated that by using cobalt ferrite–copper oxide NPs in PES-NF membranes, the Na_2_SO_4_ and NaCl rejection increased by 33% and 40%, respectively. Carbon-based NP amendments are becoming increasingly common due to their low toxicity, hydrophilic properties, and their ability to uniformly disperse in the polymer matrix [177]. Using amine- (NH_2_) functionalized CNT to modify TFC-NF membranes improved NaCl and Na_2_SO_4_ rejection [193]. CNT interlayer incorporation in TFC-NF membranes resulted in high divalent ion removal and more than 85% selectivity toward monovalent ions [194]. Introducing B-cyclodextrin into the NF membrane matrix resulted in a decrease in membrane pore size, leading to higher separation and improved permselectivity performance [195]. More than 85% of copper and lead removals were achieved by employing polyhedral oligomeric silsesquioxane (POSS) NPs and polyetherimide (PEI) based in the NF membrane [196]. MOFs are emerging NPs with tunable pore size and morphology that can be added to membrane structures to control permeability and enhance selectivity due to the dual transport routes [89,197]. Incorporation of copper benzene-1,3,5-tricarboxylate (Cu_3_(TBC)_2_) into the TFN-NF membrane improved NaCl rejection due to Donnan exclusion [198]. However, the concentration of added NPs should be optimized to avoid particle agglomeration that reduces the selectivity of NF membranes [21]. Loading high concentrations of NPs in the polymer layers might lead to an increase in void fraction of the polymer, reducing the cross-linking degree and resulting in poor separation performance. There is an upper limit for the concentration of embedded NPs, above which not only does the salt rejection sharply decrease, but also the mechanical strength of the membrane may significantly drop [179]. 

Surface modification of membranes using layer-by-layer (LBL) assembly of polyelectrolytes is a recently developed method to improve membrane selectivity [199]. Deposition of polyelectrolytes on the membrane surface affects Donnan exclusion and the pore size of the membrane, resulting in monovalent-selective NF membranes suitable for water softening [200,201]. Cheng et al., [202] demonstrated effective divalent ion removal and high selectivity of polyelectrolyte-assisted NF membranes. Modification of polyamide-NF membranes by electrolyte monomer led to improvements in the rejection of Na_2_SO_4_ and MgSO_4,_ and high selective removal of SO_4_^2−^ relative to Cl^−^ [203]. Applying UV for monomer grafting on the NF membrane is another technique to improve salt rejection in brackish water desalination [204]. However, the exposure time and the concentration of monomers play significant roles in the effectiveness of UV irradiation [204]. 

Integration with other technologies can also enhance the selectivity and performance of NF. UF-NF integrated systems demonstrated significant improvement in seawater softening [205], textile wastewater treatment [206], and drinking water purification, in terms of salts, organic compounds, and bacteria rejection, to reach the potable water standards [207]. A hybrid NF and ED system (ED-NF) has also been proposed to reach high cation fractionation in the desalination of seawater with high NaCl concentration [116,208,209]. As discussed earlier, integrating processes, even though enhances water recovery and lower fouling and scaling, increases the capital costs, energy consumption, and the operational complexity.

### 3.6. Approaches to Increase Lifetime of NF Membranes 

Membrane life span is affected by method of cleaning as well as the frequency and type of chemical agents employed. Optimization of the physical/chemical cleaning processes through the evaluation of hydrodynamics, pH, concentration of chemical cleaning solution, cleaning type, and sequence, as well as the operational conditions during cleaning, can significantly impact water recovery and salt rejection performance [157]. Chemical cleaning at high temperature is more effective and cost-efficient because of reduced chemical requirements. Chen at al. [157] identified that membrane backwashing was more efficient than forward flushing, especially when fouling occurred inside the membrane pores. Depending on the type of fouling (organic or inorganic), the pH of the cleaning solution can play a significant role in restoring membrane properties. High pH alkaline solutions provide superior cleaning for organic fouling, since they increase the electrostatic repulsive forces [210]. Acidic solutions are more efficient for inorganic scaling removal from NF membranes [157]. The effects of cleaning with NaOH and HCl on membrane performance, including the selectivity and permeability, have been studied for poly(piperazine-amide) (PPA) and PA membranes [211]. After chemical cleaning, PA membranes demonstrated superior ion rejection performance compared to PP membranes. 

In addition to proper cleaning, membrane material and mechanical strength, as well as operational conditions, play significant roles in defining membrane life span and process energy consumption. The modification of polymeric membranes by addition of aramid nanofiber that integrates easily with polymeric materials during fabrication has been recognized as a suitable approach for improving the lifetime of membranes, due to its high mechanical stability and heat resistance properties [212]. Moreover, coating the surface of the membranes with a polyelectrolyte of opposite charge improved the electrostatic bonds between the membrane polymer and coating layer, enhancing membrane durabilty during HCl cleaning [185]. 

Although polymeric membranes are the most common due to their low fabrication costs, ceramic membranes have gained increasing attention in the past two decades due to their high thermal and chemical stabilities, as well as elevated mechanical strength toward back-washing [213]. A variety of materials have been applied to fabricate ceramic membranes, including metal oxides, e.g., alumina, titania, silica, zirconia [140], zeolite, and MOFs [149]. Alumina-based membranes are more suitable for large-scale production due to their long lifetime. Zirconia-based membranes are the proper choice for the replacement of polymeric membranes under high temperature and high salinity conditions [214]. However, ceramic membranes are costly and sustain a larger pore size and less permeability compared to the polymeric membranes. These challenges have limited their use to pre-treatment processes. More research is required to further advance their application for desalination purposes [215].

### 3.7. Overall Status of NF for Brackish Water Desalination

NF has also been commercialized, and produces approximately 3% of desalinated water globally. The lower operational pressure of NF compared to RO makes it a relatively energy-efficient technique for the total desalination of low-salinity waters, or the partial treatment of high-salinity waters. NF is capable of removing multivalent ions, allowing the selective removal of scale precipitating ions and water softening [13,14]. This unique characteristic makes NF a superior option for the desalination of brackish water with low to moderate salinity that is dominated by multivalent ions [216,217]. For brackish groundwater with moderate salinity (TDS below 6000 mg/L), NF is an effective approach to produce potable water with reasonable salinity (800 mg/L) at a higher permeate flux compared to RO [14]. 

## 4. Electrodialysis

**Figure 5:**
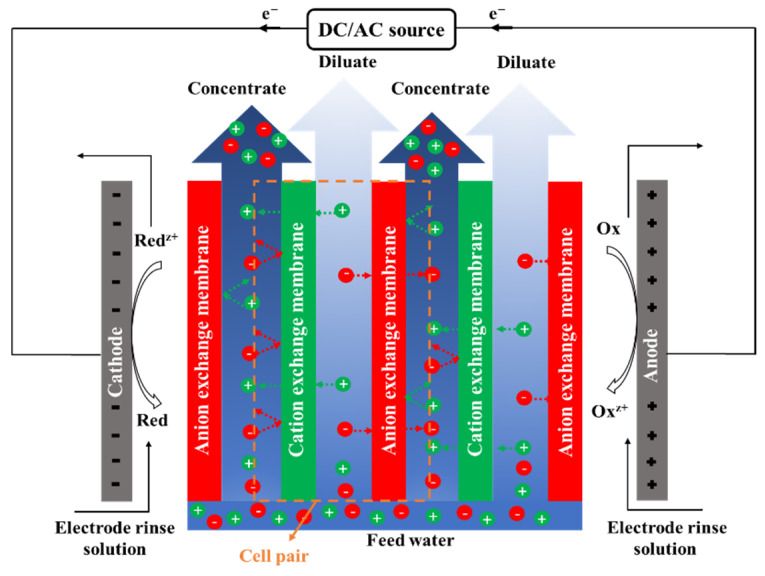
A schematic diagram of an electrodialysis (ED) stack.

Electrodialysis (ED) is an electro-membrane desalination technique that uses an electric field to separate ions from water. As shown in Figure 5, feed water enters the channels between anion and cation exchange membranes (AEMs and CEMs, respectively), alternately placed between two electrodes [218]. The imposed electric field drives ions toward opposite-signed electrodes. Cations can pass through CEM and are blocked by AEM, and anions transport through AEM and are hindered by CEM. The permselective transport of ions through IEMs results in a decrease in ionic concentrations in the diluate channels, and an increase in the adjacent concentrate compartments. An ED stack is formed by a series of repeating unit cells containing a CEM, an AEM, a diluate channel, and a concentrate channel. A typical industrial ED unit may contain over 100 cells with a membrane area of 1–2 m^2^ [218,219]. ED is traditionally designed as a plate and frame module that has low packing density [220]. A number of studies investigated designing, modeling, and optimizing a spiral wound ED (SWED) module [221,222]. The available membrane area in the spiral wound module is high due to the high packing density, decreasing the required imposed potential. Difficulties in membrane cleaning and replacement and the possibility of mixing the electrode rinse solution and water streams are the main limitations of SWED [221,222].

**Figure 6:**
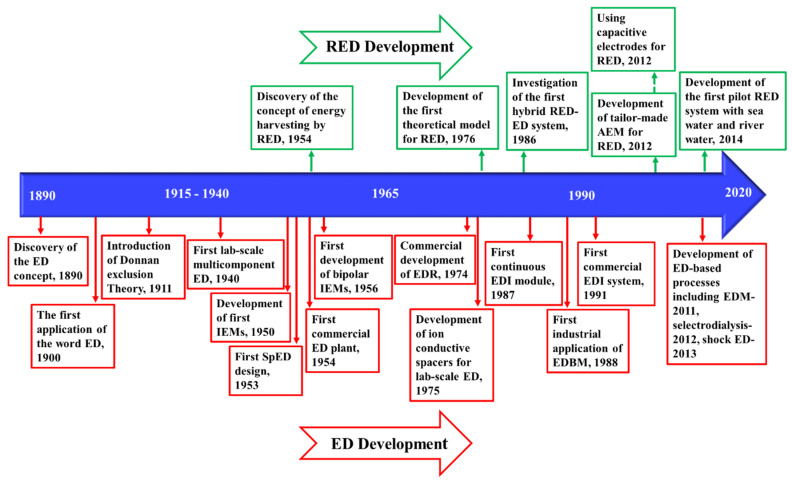
Timeline of the key developments of ED [9,121,218,221,223,224,225,226,227,228,229,230] and reverse electrodialysis (RED) [231,232,233,234,235,236,237].

Figure 6 demonstrates the timeline of the key developments of ED and ED-based processes, from the early discovery of the concept in 1890 [223], to the first commercial plant in 1954 [224], and recent advances in process development. 

### 4.1. Membranes in ED

IEMs in ED should possess high permselectivity and conductivity, low resistance, and high mechanical, dimensional, and chemical stability [215,238,239]. IEMs are made of polymeric compounds containing fixed-charge functional groups and moveable counterions. CEMs contain negative fixed charges while AEMs sustain positive fixed-charge functional groups. When CEM and AEM absorb water, the charged functional groups dissociate, leading to the release of mobile cations and anions, respectively, enabling the counterion transport through the membrane [240]. Bipolar IEMs have a cation exchange layer, an anion exchange layer, and an interfacial layer in their multilayer structure, and are used for water dissociation and acid and base production [239,240]. 

In the past few decades, numerous polymers and fabrication techniques have been developed for synthesizing and modifying IEMs [240,241,242]. A number of polymers including polyethylene (PE), polypropylene (PP), PES, polyetherketone (PEK), polybenzimidazole (PBI), polyimide (PI), poly(p-phenelene oxide), polysulfone (PSU), PEI, and polyvinylidene fluoride (PVDF) have been used in the polymeric structure of IEMs. The charged functional groups of CEMs include sulfonic acid, phosphoric acid, and carboxylic acid. For AEMs, quaternary ammonium cations, imidazole cations, and guanidinium, or nitrogen–free functional groups, e.g., phosphonium, sulfonium, and metal cations, are employed as fixed charges [243,244]. 

Properties of IEMs are governed by their material and fabrication techniques, and the type, concentration, and distribution of fixed-charge groups in the membrane structure [238,239]. Ion exchange capacity (IEC) represents the quantity of the fixed charges of IEMs, determining their permselectivity and ohmic resistance. An even distribution of fixed-charge groups in the polymeric structure results in homogenous IEMs, while an uneven distribution of fixed charges forms IEMs with heterogeneous structure [239]. In the absence of water, IEMs have a dense or non-porous structure. Once IEMs absorb water, micro-, meso-, and macro-pores can form in the swollen membranes [245]. 

Several commercial IEMs are manufactured for ED [246] and ED-related processes [247]. Typical commercial ED membranes have an ion exchange capacity (IEC) of 1–3 meq/g, an electrical resistance of 1–15 Ωcm^2^, and a transport number of 0.75–0.95 [248]. Organic and inorganic precipitation, pH changes, and the chemistry of the feed water impact the lifetime and properties of IEMs, and consequently, the separation efficiency and energy consumption of ED [249]. The development of new fabrication and modification techniques to enhance the chemical and mechanical stability, anti-fouling properties, and permselectivity of the membranes has greatly attracted the attention of the research community [240].

### 4.2. Electrodes in ED 

In ED and reverse electrodialysis (RED), which is a salinity gradient energy harvesting technology designed based on ED, electron transfer between ionic species and electrodes occurs through electrochemical (Faradaic) reactions taking place in electrode compartments. In ED, the external power supply provides the required energy for non-spontaneous redox reactions in cathode and anode compartments, while in RED, the spontaneous electrochemical reactions at the surface of the electrodes generates electricity from the existing salinity gradient between the diluate and concentrate solutions. The amount of energy dissipation in the electrode compartments depends on the electrode material, electrode design, redox couple, and supporting electrolyte solutions [250]. A number of studies have focused on optimizing and tailoring these parameters for RED and ED systems [234,250,251,252,253]. Both reactive, e.g., zinc, and inert, e.g., platinum-coated titanium, materials have been investigated for ED/RED electrodes [232,254]. Depending on the redox couples used in the electrode compartments, various electrochemical reactions can occur with the possibility of the generation of O_2_, H_2_, or Cl_2_ gases [250]. Production of H_2_, hydrogen cyanide (HCN), ClO_3_^−^, and similar compounds may impose safety, environmental, and health hazards due to their explosive or toxic nature. Moreover, potential losses in electrode compartments with redox reactions that generate gas are relatively high. However, in commercial stacks with a high number of cell pairs, electrode losses are negligible compared to the overall potential drops [250]. 

Recently, the use of capacitive electrodes has been proposed to avoid Faradaic reactions and the associated energy dissipation and hazardous byproduct generation [236]. Capacitive electrodes contain a current collector and an activated carbon layer, where ions are stored in the electric double layer (EDL) and balanced out by electronic charges on the electrodes. Capacitive flow electrodes (FEs), generally composed of micro- or nano-particles of carbon in an electrolyte solution with a weight percentage below 25%, have also been investigated for RED [255]. The improved performance of RED using FEs offers a possible application of this type of electrode in ED as well.

### 4.3. Operational Modes of ED

ED is operated in continuous, batch, and feed-and-bleed modes [256]. The continuous mode is suitable for a large-scale ED, while the batch mode is appropriate for small- or medium-scale processes [257]. In the feed-and-bleed mode, the desalinated or concentrate streams are partially recycled back to the feed solution to control the salinity of the product water [258]. This operating mode results in a higher water recovery and is applicable to medium- and large-scale processes [218,258]. These processes are operated at either constant potential or constant current density [259].

**Figure 7:**
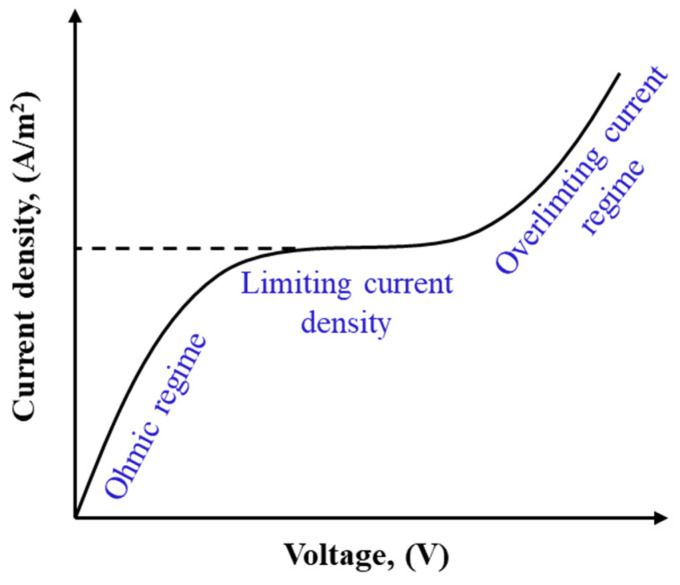
A sketch of the current density–voltage curve in ED.

ED is operated at sub-limiting, limiting, and over-limiting current regimes as illustrated in Figure 7 [260]. In sub-limiting regimes, current density increases linearly with imposed potential. Due to the permselectivity of IEMs, CP forms in channels and develops along the cell. Once the concentration of ions at the membrane–solution interface in the diluate channel approaches zero, the current density reaches a limiting value, shown as a plateau in Figure 7. Further increasing the cell potential beyond the limiting value promotes a series of phenomena including gravitational convection, water splitting, co-ion leakage, and electroconvection in the cell, leading to the over-limiting of the current density and a transition to a linear increase in current density with cell potential [261,262]. The enhanced turbulence in the over-limiting current regime may result in mechanical instability. Additionally, a higher degree of water dissociation at higher potentials may lead to drastic pH changes, increasing salt precipitation and membrane scaling. ED units are typically operated at 80% of the limiting current density to avoid such consequences and to control the operational energy costs [263]. 

Operating ED at an over-limiting current regime increases the rate of mass transfer and allows a decrease in the required membrane surface area, reducing the cost associated with IEMs [262,264]. However, economical operation of ED at an over-limiting current regime depends on the membrane properties. To reach the over-limiting current conditions at lower potentials and reduce the energy requirements, the membrane physical and chemical structure should be modified. Electroconvection can be enhanced by increasing the density of membranes’ charged groups, as well as the surface heterogeneity and hydrophobicity [262,265,266]. Water dissociation (pH changes) at over-limiting currents can be controlled by developing membranes that have homogeneous charge distribution [267] and contain fixed charges with lower catalytic activity for the water splitting reaction [268]. Although increasing membrane surface heterogeneity enhances the electroconvection, it is normally accompanied by higher water dissociation as well as fouling and scaling formation. Hence, care must be taken in the operation of ED with heterogeneous membranes at over-limiting current regimes for water with a high fouling propensity. Studying the over-limiting current operation of ED for various feed waters [269,270], investigating the effects on transport mechanisms [271,272], and identifying approaches to modify membrane characteristics [273,274] are ongoing research directions. 

### 4.4. Development of ED-Based Processes

Since the first commercial ED plant in the 1950s, a number of ED-based processes have been developed, including electrodialysis reversal (EDR) [275], bipolar membrane electrodialysis (EDBM) [276], electro-deionization (EDI) [277], electrodialysis metathesis (EDM) [278], and RED [279]. In EDR, the polarity of the power source is periodically reversed at intervals between a few minutes to a few hours, reversing the ionic flow direction. EDR is a commercialized process with a lower fouling and scaling potential and higher water recovery compared to ED. EDBM takes advantage of bipolar membranes and converts salts into acids and bases. In EDI, the diluate channel is filled with ion exchange resins to improve the conductivity of the solution and avoid the back-diffusion of ions from the concentrate channel. Water dissociation in the diluate channel can result in the self-regeneration of ion exchange resins and aid in the continuous operation of the process. EDI is used for ultra-pure water production. EDM is a novel alteration of ED in which metathesis reactions take place to convert less soluble salts to more soluble species. The repeating unit cell contains four compartments (quad) with two diluate and two concentrate channels. Feed water enters one of the diluate channels and the substituting solution enters the other. Non-precipitating salts are formed in the concentrate compartments. In EDM, high water recovery can be reached, decreasing the volume of the concentrate waste stream and enhancing the ability to produce salts. RED is a technique to harvest salinity gradient energy (blue energy) from the mixing of two streams with different salt content [279]. Blue energy is not affected by seasonal changes, unlike solar and wind, and can serve as a source of renewable energy. The development of these ED-based processes can broaden the applications of the processes for a wider range of feed water quality. However, further research should be conducted on these novel processes to improve the operational conditions, as well as on the membrane and electrode materials to enhance their energy- and cost-effectiveness. 

### 4.5. Main Challenges in ED

Desalination efficiency in ED is affected by the performance of IEMs, fouling and scaling, and the electrical resistance of the cell. We summarize the research and development conducted, to overcome such challenges and improve the performance of ED. 

### 4.6. Approaches to Improve Performance of IEMs in ED

The economics and energy consumption of ED depend on the cost and selectivity, conductivity, and stability of IEMs [264]. Traditional fabrication techniques used for synthesizing homogeneous IEMs include the direct polymerization of monomers that contain ionizable groups, grafting charged functional groups onto polymeric film, and introducing charged groups to the polymer followed by dissolving it in an organic solvent and casting the solution on a plate. Ran et al., [240] reviewed emerging fabrication techniques used for synthesizing IEMs, including polymer blending, pore filling, pore soaking, in situ polymerization, and electro-spinning. In the polymer blending method, several polymers are mixed to overcome the deficiencies of a single polymer and produce membranes with higher selectivity and conductivity. Pore filling and pore soaking approaches result in IEMs with high permselectivity and low swelling. In situ polymerization reduces the amount of toxic organic solvents used in traditional fabrication approaches. The IEMs synthesized through electro-spinning sustain high porosity and a high specific surface area. Although promising, further investigation is required to optimize the mixing ratio in polymer blending, improve the long-term stability of IEMs generated through pore filling, and extend the application of electro-spinning for synthesizing IEMs from various polymers in a larger scale [240]. 

In addition to investigations of new fabrication techniques for IEMs, numerous researchers have focused on modifying the chemical and physical properties of the existing IEMs. Various NPs, e.g., zeolites, carbon-based, graphene-based, silica, titanium oxide, silver, aluminum oxide, etc. have been used to produce nanocomposite IEMs [248]. The addition of NPs to IEMs enhances membrane properties due to the addition of functional groups of NPs or the dispersion of ionic clusters inside the polymeric structure of the membranes. However, the optimization of the appropriate type and quantities of NPs is essential to avoid shielding the charged functional groups of IEMs and causing mechanical instability [248]. 

Chemical and physical modifications of membrane surfaces are alternative approaches to improve their permselectivity and antifouling properties [280]. Surface modification techniques such as plasma treatment, adsorption, solution casting, ion implantation, and polymerization adjust surface properties either by coating a layer on top of the surface or by direct improvement of the chemical structure of the surface. These approaches aim to modify surface wettability, smoothness, homogeneity, charge density, anti-bacterial properties, and multivalent ion rejection. For long-term effectiveness of these approaches, the modified surface should be durable and stable.

Many researchers have focused on the development of IEMs with high counterion permselectivity or high specific ion selectivity, e.g., monovalent or nitrate permselective IEMs [281]. Permselectivity of IEMs is impacted by the affinity of ions with membranes and their mobility inside them [282]. A variety of approaches are used to improve permselectivity, including [281] surface modification through developing a highly cross-linked surface layer (to enhance the steric sieving effects), coating a thin oppositely-charged layer on the surface of the membrane (to provide higher electrostatic repulsion for multivalent ions), layer-by-layer film deposition (to provide higher rejection toward multivalent ions due to the increased Donnan exclusion effects and increased hydrophobicity of the surface), and coating a dense and non-charged polymeric layer on the surface of IEMs (to increase size sieving effects). Enhancing IEM properties through advancing fabrication and modification techniques is an ever-growing research direction that can significantly affect desalination performance in ED and related processes.

### 4.7. Approaches to Reduce Fouling and Scaling in ED

ED, similarly to the pressure-driven processes, suffers from membrane fouling and scaling. Fouling occurs due to size exclusion of particles, electrostatic interactions of foulants with charged functional groups of the IEMs, hydrophobic interaction of the organic foulants with uncharged sections of the IEMs, microbial activity, and salt precipitation and deposition [283,284]. Mineral scaling is mainly dominant on the CEM (due to the precipitation of salts such as Ca(OH)_2_, Mg(OH)_2_, and CaCO_3_) while organic and colloidal fouling mainly occur on the AEM (due to the negative charges of such organic compounds causing electrostatic adsorption to the positively charged membrane surface) [285,286]. Parameters affecting fouling and scaling include size and concentration of foulants; salinity, composition, temperature, and pH of water; operational mode of the process (ohmic, limiting, or over-limiting current regimes); cell hydrodynamics; and membrane properties including morphology, pore size, and chemistry [284]. Fouling and scaling lead to a decrease in IEM permselectivity and an increase in membrane ohmic resistance [249,283]. 

Similar to RO and NF, pre-treatment steps such as filtration, pressure-driven filtration [287], activated carbon [288], pellet reactor [289], UV irradiation [290], and phytoremediation [291] have been shown to reduce fouling and scaling issues. However, the addition of the pre-treatment steps increases the capital costs and operational complexity. Chemical and physical cleaning of the IEMs are used to remove fouling and scaling and restore membrane properties. Cleaning methods are generally the same for pressure-driven processes and ED. However, a number of mechanical removal techniques such as backwashing, air sparging, and forward flushing that are applicable to pressure-driven processes may damage IEMs, due to their non-porous structure and ability to pass ions rather than water [284,292,293]. The choice of cleaning agents depends on the structure of the membranes as well as the nature and intensity of fouling [293,294]. Guo et al., [295] identified that HCl is a superior agent for removal of CaCO_3_, BaCO_3_, or Mg(OH)_2_ scale, while NaOH is more efficient in the elimination of organic and oily foulants. It is worth noticing that chemical cleaning with some strong oxidizing or alkaline agents may result in the deterioration of charged functional groups or the polymeric matrix of the IEMs [296,297]. In addition, the chemical cleaning results in the production of an effluent waste stream which needs to be properly managed. The cost of chemical agents further increases the cost of desalination. 

Membrane modification is also employed in ED as an anti-fouling approach. Many of the modification techniques used to improve IEM antifouling are similar to those for pressure-driven membrane processes. Physical and chemical alterations of the membrane surface are used to adjust surface charge density, increase the hydrophilicity, and reduce the roughness. Increasing the negative charge density and hydrophilicity of the surface through coating a polyelectrolyte or a thin nanocomposite layer on the surface has enhanced the antifouling properties of the membranes [298,299,300]. However, these surface coatings may result in an increase in surface roughness, adversely affecting AEM fouling resistance [285]. Hence, the concentration of the coating layer and the fabrication time and conditions should be optimized to minimize fouling. Approaches to control biofouling typically focus on preventing the attachment of microbial communities to the surface of the membrane, or destroying bacterial communities adhered to the surface. Such anti-adhesion and anti-microbial approaches are achieved through modification of the membrane by coating a layer of polyelectrolyte or silver nanomaterials on the surface [301,302,303]. 

A unique advantage of ED over RO and NF is the possibility of manipulating the operational conditions to mitigate fouling and scaling. Periodically switching the polarities of the electrodes in EDR reduces fouling and scaling by detaching organic foulants and dissolving deposited minerals [284]. A single-pass EDR with no anti-scalant addition was able to achieve high water recovery for feed water with a CaSO_4_ saturation level above 190% [304,305]. By using modified thin spacers and high diluate flow rate relative to that of the concentrate stream, Turek and Dydo [304] reached more than 90% water recovery in an EDR of feed water supersaturated by CaSO_4_ and CaCO_3_. Pulsed electric field operation of ED (PEF-ED) is an alternative low-maintenance approach to control fouling and scaling and minimize chemical dosing [306,307,308,309,310,311,312,313,314,315,316,317,318]. PEF-ED consists of pulse periods with a constant electric field followed by pausing lapses with no imposed electric field [319]. PEF-ED leads to the restoration of concentrations in the boundary layers during the pause lapses, minimizing CP in the cell [306,318]. Hence, PEF operation can reduce water dissociation, fouling and scaling, and energy inefficiency in ED. In current studies pulsing parameters (frequency and duty cycle) are selected randomly and different values are reported for various feed water compositions. To reduce the number of trial steps and maximize the performance of the PEF-ED, a systematic approach should be developed for optimizing the pulsing parameters according to the chemistry of the feed water.

### 4.8. Approaches to Decrease the Electrical Resistance in ED 

Decreasing the electrical resistance of an ED cell minimizes energy dissipation and improves ion separation efficiency. Modifications of chemical and physical structures of membranes to reduce electrical resistance and improve the mixing at the surface of the membranes can improve the electrical conductivity of the ED unit. Increasing the concentration of charged functional groups on the membranes decreases their ohmic resistance. However, increasing IEC is normally accompanied by higher water uptake in the membranes and lower dimensional stability [320]. The balance between IEC and water uptake of the IEMs is achieved through cross-linking methods [321,322,323]. The conductivity of the cross-linked membranes is controlled through adjusting the cross-linkers type, cross-linking process, time, and temperature [324].

Corrugated or profiled membrane surfaces are an alternative approach to improve the conductivity of ED. The use of profiled IEMs eliminates the need for a non-conductive spacer in the channel that can reduce membrane area (shadowing) and increase resistance. Pawlowski et al., [325] reviewed the development of profiled IEMs, their application in electro-membrane processes, limitations, advantages, and the preparation techniques. Using profiled IEMs and a high feed water flow rates increases turbulence in the channel, improving cell conductivity and ion transport. Under such conditions, fouling and scaling are minimized which further decreases the electrical resistance of ED. Due to better mixing with profiled membranes, CP and water dissociation are less significant, resulting in an improvement in current efficiency. Furthermore, since fouling and scaling increases electrical resistance, fouling mitigation approaches also help minimize the ohmic resistance.

In addition to membrane modification, improving the properties of spacers can significantly help reduce the cell resistance. Spacer nets are used inside the channel to keep membranes separated and promote cross-channel mixing, minimizing CP formed as a result of IEM’s semi-permeability. Reducing CP increases the conductivity in the boundary layers of the diluate channel, enhancing the current efficiency. Geometry, water contact angle, mesh size, and filament size of spacer screens should be adjusted to enhance their performance and reduce the corresponding pressure losses in channels [326,327,328,329]. Although polymeric spacer nets increase the conductivity of the boundary layer, they may increase the overall electrical resistance due to their non-conductive nature, shadow effects, and fouling potentials. Ion-conductive spacers were developed to reduce the electrical resistance of the spacers and increase ion transport and current density [330,331,332,333]. Despite such advantages, ion-conductive spacers have not been adopted in commercial ED plants, possibly due to their complexity and high production costs as well as the increased fouling potential due to surface charges [218,284]. More recently, Balster et al., [334] proposed the use of gas sparging as an alternative to spacer nets to promote mixing in the spacer-free channel, and were able to increase mass transport with minimal increases in cell resistance. Mechanical stability of the membranes in such spacer-free channels and means of achieving uniform distribution of the air bubbles require further investigation [334]. 

### 4.9. Status of ED for Brackish Water Desalination

ED and ED-based processes (EDR and EDI) produce about 3% of the global volume of desalinated water [7]. ED has been found to provide cost-effective desalination of brackish water with a salinity between 1000 and 10,000 mg/L [264]. At water recovery above 80%, the energy requirement of EDR is lower than continuous RO and semi-batch RO for the same salinity of the feed water [335]. The ability to operate at low-pressure and with less pre-treatment due to the more robust nature of IEMs compared to RO membranes is among the main advantages of ED. 

## 5. Membrane Capacitive Deionization

### 5.1. Capacitive Deionization 

**Figure 8:**
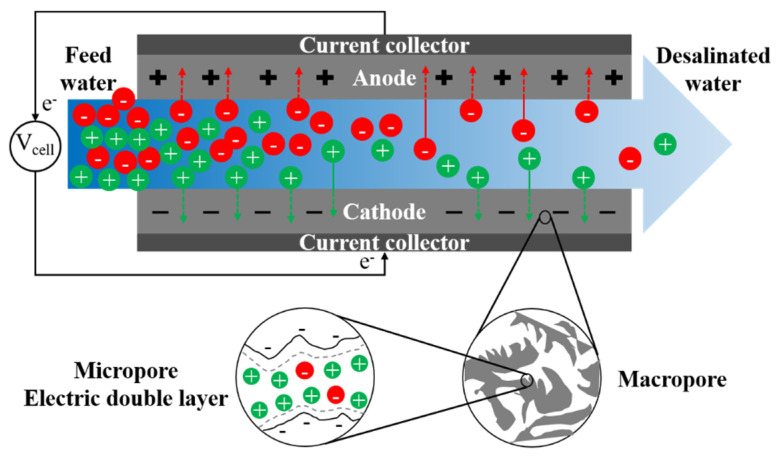
A schematic graph of the desalination process in conventional capacitive deionization (CDI).

Conventional capacitive deionization (CDI) uses porous electrodes to extract ionic species from feed water via electro-sorption [336]. Ion adsorption in electrodes occurs due to electrostatic forces between applied electronic charges on electrodes and ionic species in water. During adsorption, there is no charge transfer between electrode and electrolyte, and the mechanism of ion adsorption and storage is analogous to that of a capacitor. The capacitive electrode is often assumed to be composed of both micropores, where ions are stored forming an electric double layer (EDL), and macropores, which provide the transport path from the bulk fluid (Figure 8) [337]. Non-conductive spacers are typically used in channels between electrodes to prevent electrical short circuiting, increase mixing, and reduce formation of CP at the electrode-solution interface [338]. Mosaic membranes [339], ion-exchange resins [340], and granular activated carbons [341] have been applied as spacers in MCDI to reduce cell resistance.

During desalination (when electrical potential is applied to the electrodes), ions are attracted toward oppositely charged electrodes and are adsorbed (Figure 8) [342]. During electrode regeneration, the electrodes are short-circuited or their polarity is reversed to repel ions back into the solution, generating a concentrate stream [342]. The electro-sorption performance during the desalination cycle depends on applied voltage, flow rate, cell configuration, desalination/regeneration cycle times, as well as the physiochemical properties of the electrode [343]. 

### 5.2. Membrane Capacitive Deionization 

**Figure 9:**
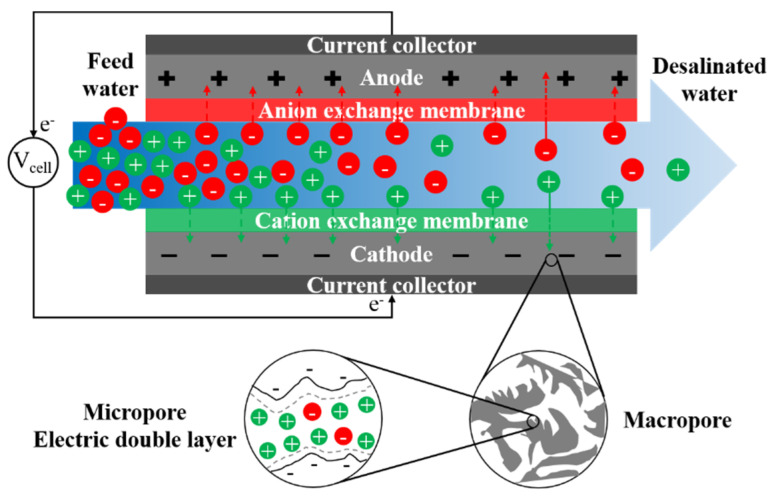
A schematic graph of desalination process in membrane capacitive deionization (MCDI).

Membrane capacitive deionization (MCDI) is a modification of conventional CDI by incorporating IEMs between the electrode and spacer. A CEM is attached to the cathode and an AEM is attached to the anode. The MCDI cell assembly and ion transport route during the desalination process are described in Figure 9. The role of IEMs is to prevent co-ions from being flushed out of electrodes during desalination, or penetrating into electrodes during regeneration. Thus, MCDI improves charge efficiency, salt adsorption capacity, desalination rate, and energy efficiency compared to conventional CDI [338,344].

**Figure 10:**
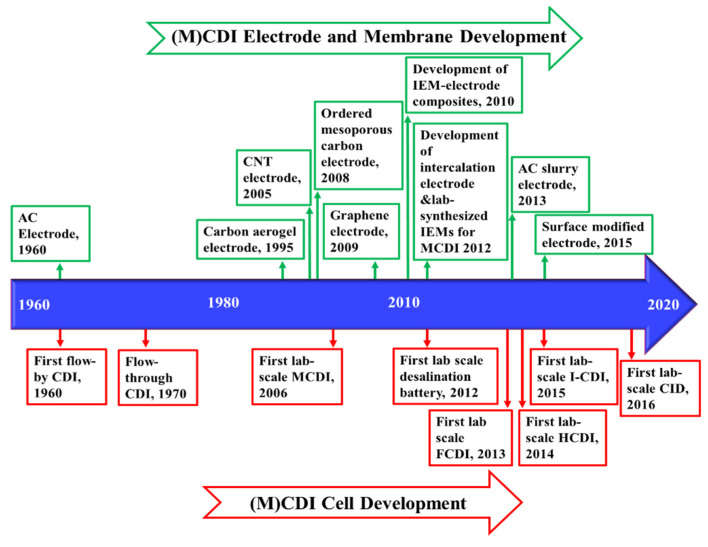
Timeline of the key developments of the cell structure [345,346,347,348,349,350,351,352], membranes, and electrodes of (M)CDI [352,353,354,355,356,357,358,359,360].

The first MCDI cell was developed in 2006 [345], a few decades after the first design of CDI [346]. More recently, electrode and membrane materials and cell structures have been improved in MCDI as summarized in Figure 10 and discussed below. These developments remain at bench and pilot scale, but the relatively low energy consumption suggests that scale-up and commercialization may be feasible. 

### 5.3. Membranes in MCDI

Commercial IEMs with high permselectivity as well as high chemical and mechanical stabilities are appropriate for MCDI [361]. MCDI does not require self-standing membranes, thus, thinner IEMs and electrode-IEM composites with relatively low electrical resistance and favorable stability have been developed for MCDI. Fabrication techniques including solution casting [362,363,364] and pore-filling polymerization [240,365,366,367,368] are employed to synthesize thinner and more conductive IEMs, specifically for application in MCDI. IEMs synthesized via casting methods possess low resistance [362,363,364]. Membranes fabricated through pore-filling techniques have shown superior dimensional and chemical stabilities [365,366,367,368]. The γ-irradiation process during pore-filling polymerization was proven to help further strengthen membrane dimensional stability and anti-chemical corrosion resistance. Kim et al., [367] fabricated a CEM through pore-filling with both sulfonic acid groups and weak acid chelating groups and the resulting IEM was stable over a wide range of pH and was effective for multivalent cation removal. In addition, electrode-IEM composites are produced by immersing or spraying the electrode with functional solutions, in situ polymerization of the electrode, and electrode chemical oxidation with dopant [369]. Electrode-IEM composites possess low contact resistance at the interface of the electrode and ion exchange polymer, as well as enhanced capacitance in some cases [361,370]. Electrode-IEM composites synthesized via IEM blending with carbon slurries possess relatively low material cost [371]. Despite these advances, the stability and longevity of the IEMs in MCDI require further improvements to fully develop the technology [371]. Fouling issues and the appropriate cleaning methods of these electrode-IEM composites should also be explored.

### 5.4. (M)CDI Operational Modes

(M)CDI (refers to both CDI and MCDI) is operated under constant voltage (CV) or constant current (CC) modes [372,373,374]. Under CV mode, the effluent concentration decreases rapidly but sorption of ions slows as the electrodes approach capacity. Under CC mode, the concentration drops to a minimum value and remains constant until the end of the desalination cycle. Saleem et al., [375] operated a CDI cell under the hybrid CV-CC mode, achieving a low concentration rapidly under CV mode first and switching to CC mode to maintain the concentration. The hybrid CV-CC mode improved ion removal compared to CC and CV modes.

CC mode generally consumes less energy compared to CV mode over complete cycles. However, the energy consumption of MCDI in a batch process under various operational modes largely depends on the concentration of feed water, concentration of the product water, and the volume of the product water in a batch mode MCDI [376]. At the same concentration of feed water, energy consumption of both modes increases at higher water production rates and lower product concentration. At the same adsorption objectives and salt removal rate, CV mode exhibits a smaller change in SEC compared to CC mode when doubling the volume of product water or lowering the salt concentration of product water. However, Dykstra et al., [377] identified that under the same desalination objectives, CC mode could recover more energy. Kim et al., [378] demonstrated lowered energy consumption by marginally charging the electrodes (0.3 V) during regeneration in CV mode, as a result of a higher charge efficiency. Further study should be conducted on the energy consumption of CC and CV modes at the same cell size, feed water quality, water recovery, salt removal efficiency, and energy recovery mode to make a valid comparison of these operational conditions. 

### 5.5. CDI Cell Architectures

**Figure 11:**
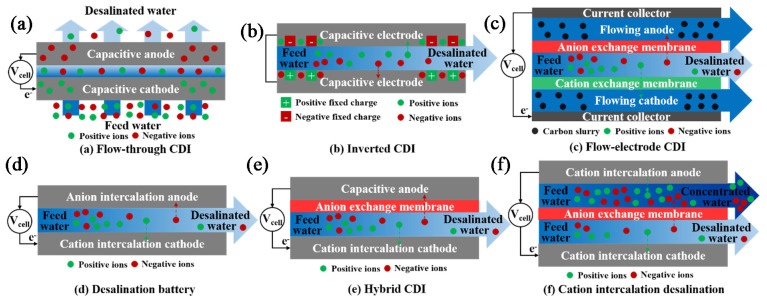
Cell architectures: (**a**) flow-through CDI; (**b**) I-CDI; (**c**) FCDI; (**d**) desalination battery; (**e**) HCDI; (**f**) CID.

A variety of cell architectures have been developed for CDI. In addition to conventional CDI and MCDI, there are flow-through CDI, inverted CDI (I-CDI), flow-electrode CDI (FCDI), and CDI with intercalation electrodes (also known as Faradaic electrodes) which includes hybrid CDI (HCDI), and cation intercalation desalination (CID) and a battery architecture (Figure 11). Here, we provide a summary of various designs of (M)CDI-based processes along with their main merits and drawbacks and most recent research studies that have improved their performance in Table 2. However, a more detailed review was conducted by Tang et al., [379]. 

Modifications of the cell structure and electrode materials, as well as the efforts to change configurations of (M)CDI, are aimed at decreasing energy consumption and increasing the adsorption capacity. These modifications can help make (M)CDI processes more competitive with RO in the desalination of low-salinity water.

### 5.6. Energy Recovery in (M)CDI

During the regeneration step, ions are repelled from the electrode by reversing the polarity or by the short-circuiting of electrodes. The energy stored in EDL during desalination can be partially recovered during regeneration. Direct energy recovery in (M)CDI can be implemented via a buck-boost converter to transfer part of the stored energy which is released during regeneration to an inductor and later discharge it to a capacitor or another (M)CDI cell [393]. The amount of energy recovery in (M)CDI is affected by the operational mode, applied current and voltage, salinity of the feed water, salt removal capacity of the electrodes, and cell hydrodynamics [333,394,395,396,397]. The energy recovery ratio (the energy recovered over the energy consumed) of CC mode is higher than that of CV mode under the same discharging mode [394,395]. During regeneration, increasing the applied current or voltage negatively affects the energy recovery due to higher energy dissipation through cell internal resistance and the ohmic resistance of the external load [333,394]. Salt removal capacity has a positive impact on energy recovery since greater salt removal increases reversible electrical energy storage [395]. Higher salinity of the feed water and a thinner channel result in greater energy recovery due to the relatively low resistive energy loss under these conditions [396]. An energy recovery system has also been widely applied in various FCDI layouts. Ma et al., [398] recovered energy with a two-chamber device by applying reversed polarity on an isolated circulating flow-electrode and inserting an AEM to eliminate short-circuiting. Porada et al., [399] incorporated a pair of cylindrical IEMs into FCDI as pathways for the saturated flow-electrodes to continuously harvest energy based on the principles of capacitive mixing to generate energy from the salinity gradient and gas phase CO_2_ gradient. Energy recovery in FCDI could be promoted by increasing the electrolyte concentration, enhancing the content of the flow-electrode, and adding conductive additives [398,400].

### 5.7. Main Challenges of (M)CDI

Although (M)CDI has been investigated for a wide range of applications, a series of challenges still exist. Limited electrode capacitance, high electrical resistance of the cell elements, fouling and scaling, and irreversible Faradaic reactions are among the key challenges that should be addressed to further advance the MCDI technology. Current efforts in these areas are discussed below.

### 5.8. Approaches to Improve Electrode Performance

Ion removal efficiency in (M)CDI is influenced by electrode surface area, pore geometry, surface charge, and conductivity of the electrode, as well as by the imposed electric field [401]. The adsorption capacity of (M)CDI is mainly controlled by the physiochemical properties of the electrode materials and the applied voltage. Charge efficiency, which is defined as the ratio of ion adsorption at equilibrium to the applied charge [402], depends on the extent of co-ion repulsion and the occurrence of unexpected side reactions, e.g., oxygen reduction [403]. Charge efficiency is one of the key factors for evaluating electrode performance. Each imposed electronic charge is supposed to remove one salt ion from the solution. In reality, the applied charge is used to adsorb counterions as well as to reject co-ions [378]. Lower charge efficiency results in higher energy consumption. Grafting ion-selective functional groups onto electrodes [404,405] can reduce co-ion repulsion and increase charge efficiency. During desalination, IEMs in the MCDI cell prevent co-ions from being repelled out of the electrode, improving the charge efficiency in the process. Intercalation electrodes also help adsorb counterions more efficiently. In many cases, charge efficiency and adsorption performance can be improved in a similar manner.

As mentioned above, the adsorption capacity of the electrodes and the ion removal efficiency in (M)CDI can be improved by tuning the pore geometry. A high specific electrode surface area is usually preferred to increase ion adsorption; however, it does not necessarily ensure better performance due to the potential for dead-end or poorly interconnected pores [406]. These unfavorable pore geometries hinder the diffusion of ions inside pores and thus reduce electro-sorption. Various parameters, e.g., precursor loading, calcination temperature, heating–cooling rate, and different synthesis routes are being used for tuning pore geometry [407]. The micro/mesopore ratio also plays an important role since micropores (<2 nm) ensure a large adsorption surface area while mesopores (2–50 nm) [408] facilitate quicker transport of salts to and from sorption sites [409]. The efficiency of both adsorption and desorption are important to overall performance. Chmiola et al., [410] showed increased volumetric capacitance for pore sizes smaller than the solvated radius (less than 1 nm). The optimum pore size range for ion adsorption in EDL in CDI was reported as 0.8–2 nm [411]. However, this optimum pore size is still under debate. If the thickness of the EDL is similar to the pore width, the double layers formed on the pore walls overlap, which hinders ion adsorption inside the pores. Yang et al., [412] introduced the concept of a cut-off pore width (0.6 nm), below which there is no effective ion adsorption inside the pore. However, the thickness of EDL depends on the ionic concentration and applied voltage [413]. The overlapping effect is reduced at a higher imposed voltage and salt concentration, ultimately resulting in better adsorption for a small pore size [414]. In addition to pore size optimization, current research is focused on controlling pore structure by using various calcination environments, e.g., ammonia [415], developing hierarchical porous biomass-derived carbon materials [416,417,418], and using MOF-derived materials [419,420] and biomass-derived materials [416,421,422]. The high surface area and tunable pore size of MOFs can be utilized to prepare electrode materials. Large-scale production and mechanical stability of these MOF-derived materials under long-term cyclic operation should be further studied to advance their application in electrode materials.

Another approach to improve the ion adsorption capacity of electrodes is material doping, which helps rearrange charge distribution and adjust the electronic properties of the electrode. Doping can be done through using precursor materials containing the dopant molecules, adding an external element containing the dopants, or treating the material in an environment that introduces the dopants into it. While nitrogen doping of carbon materials is widely used [423,424], other atoms, e.g., phosphorus [425] and sulfur [426], can also improve conductivity and wettability by the formation of defects on carbon materials. The outermost shell electron configuration of phosphorus is similar to nitrogen, while its larger diameter enables the creation of more deformations in carbon atoms [425]. Larger atoms are capable of better improvement of electrical conductivity by imposing more polarization in the electric field and creating more charge positions [427]. Multiple atomic doping can promote synergistic effects, resulting in better conductivity and hydrophilicity that ultimately results in improved ion adsorption [426,428]. While multiple atomic doping results in improved conductivity, this technique often suffers from an increased dopant leaching effect. Synthesis processes need proper optimization to take advantage of multiple atomic doping. The improper distribution of dopant atom in the electrode material is another limitation which should be minimized.

High hydrophilicity of the electrode materials ensures complete wetting by the aqueous solution, further increasing the electro-sorption of ions and enhancing adsorption capacity. Introducing polar functional groups to the electrode materials significantly reduces the surface contact angle of the electrode, making it more hydrophilic [429]. Cheng et al., [430] recently reviewed various modification strategies including coating, heteroatom doping, and functionalizing the electrode surfaces to improve the specific capacitance, conductivity, and hydrophilicity of the CDI electrode. Several strategies including insertion of multiple wall CNTs [431], acid treatment [46,432], base treatment [433,434], oxygen plasma treatment [435], and heteroatom doping [427] to introduce surface functional groups [406] are reported to improve the hydrophilicity of the electrodes. Grafting sulfonic and amine functional groups into the electrode materials was reported to increase hydrophilicity as well as the selective adsorption of ions from saline water [436,437]. Although these strategies improve the hydrophilicity and ion adsorption capacity of electrode materials, their deficiency in non-charged pollutant removal puts limitations on the performance of (M)CDI processes and requires further investigation.

An alternative approach to overcome the finite adsorption capacity of carbon electrodes is utilizing intercalation electrodes with Faradaic reactions to capture ions from saline water [438]. Yu et al., [439] reviewed various types of intercalation electrodes with different deionization cell configurations and discussed material properties for such electrodes. The ion adsorption capacity of intercalation electrodes is much higher than EDL capacitance. In capacitive electrodes, the applied charge is partially used to repel co-ions from the electrode, which results in low charge efficiency. Electrodes involving Faradaic reactions might be asymmetric [440] to provide better oxidation and reduction function to capture cation and anions from saline water [441]. Intercalation electrodes [442,443] increase counter-ion adsorption and thus improve charge efficiency. Suitable crystal structure [444] and proper interlayer spacing [441] facilitate rapid diffusion of ions inside intercalation electrodes. Incorporating organic materials into intercalation electrodes [73,442] helps leverage their properties, e.g., high specific capacity. A tremendous number of research studies are devoted toward the development of EDL-based conventional carbon electrode materials, as well as Faradaic electrodes. Carbon-based electrode-related research is focused on different strategies to improve ion adsorption capacity and charge efficiency, which will provide durable electrodes with appropriate porous structure. The theoretical molecular simulation studies on electro-sorption physics facilitate the research efforts on tailoring electrode material more effectively. Along with the need to develop better materials, proper characterization of the physicochemical properties of electrodes and the evaluation of their ion adsorption performance is essential. Faradaic electrodes show promising performance in regards to improving ion adsorption capacity, but still more research is required on other aspects of the (M)CDI processes, including the cell architecture, operational conditions, and economic analyses to further advance the industrial application.

### 5.9. Approaches to Decrease Fouling and Scaling in (M)CDI

The extent of fouling in CDI and MCDI is different due to the existence of IEMs in the latter. Fouling in CDI mainly occurs on electrodes and results in a reduction in electrode conductivity, electro-sorption capacitance, and cell stability. Organic foulants reduce cell performance by blocking electrode pores, competing with ions for adsorption sites, hindering ion diffusion to the electrodes, and accelerating intercalation electrode dissolution [445,446]. The observed scaling issues are less severe than fouling in (M)CDI studies, which may be a result of the relatively low hardness of brackish water used in (M)CDI. The alternating desalination and regeneration processes also help to reduce the accumulation of scale. Zhang et al., [447] showed that the effects of calcium and magnesium scale on the long-term performance of CDI cells were limited due to the sufficient desorption of these scale-forming ions from electrodes during regeneration. Silica stays neutral during CDI operation and does not contribute to scaling. Ferric ion intensifies electrode scaling due to the formation of iron hydroxide deposits on the surface of the electrode [445,448].

Electrode modification techniques are used to reduce the extent of fouling in CDI. TiO_2_-coupled electrode composites possess anti-fouling properties due to their photocatalytic ability, removing HA foulants [449]. Zwitterionic polymer molecules coated onto carbon electrodes mitigated fouling by improving hydrophilicity of the electrode surface and preventing organic foulants from reaching the electrode [450]. A dual-layer electrode constructed from attaching an ultrafiltration membrane onto carbon was able to keep the organic foulants from reaching the electrode and used an electro-catalytic oxidation reaction to remove the foulants from the solution [451]. Electrode modification for reducing fouling issues is an active research area. The observed stability of the modified electrode and the effectiveness of antifouling confirm the practicability for future industrialization. Future research should be conducted to evaluate the cost effectiveness and operational challenges of CDI with modified electrodes in large-scale.

To regenerate the fouled electrodes, hydraulic cleaning as well as acid and alkali cleaning are applied to remove foulants and scales from electrodes. An acidic solution (0.1 M hydrochloric acid) and an alkali solution (0.1 M sodium hydroxide) are used to remove accumulated scale and foulants, respectively [448]. Although alkali cleaning could effectively remove most organic foulants, it could alter the electrode surface structure, accelerating Faradaic reactions, and lead to electrode corrosion for electrodes containing PVDF binder [446]. Pre-treatment steps would then still be necessary to lower dissolved organic compound content in feed water to sustain cell performance.

In MCDI, electrode fouling has been found to be less severe than that in CDI due to the presence of IEMs [446]. However, IEMs may still clog, resulting in an overall reduction in cell performance [452]. The period of desalination in MCDI should be kept sufficiently short to avoid penetration of the foulants through IEMs. Even though the scale formation in MCDI is negligible, minerals can increase the formation of organic fouling [452,453]. Surface modification of IEMs has been investigated as an antifouling approach for MCDI [284]. Fouling on IEMs can be removed by the reverse polarity during MCDI regeneration [446]. Mild alkali solution has been used to remove organic foulants from IEMs [284,452]. AEMs accumulate most of such foulants [446] but AEMs do not maintain their integrity in high pH alkali solutions. As a result, pre-treatment for the removal of dissolved organic compounds is suggested for the sustainable operation of MCDI.

### 5.10. Approaches to Minimize the Irreversible Faradaic Reactions

**Figure 12:**
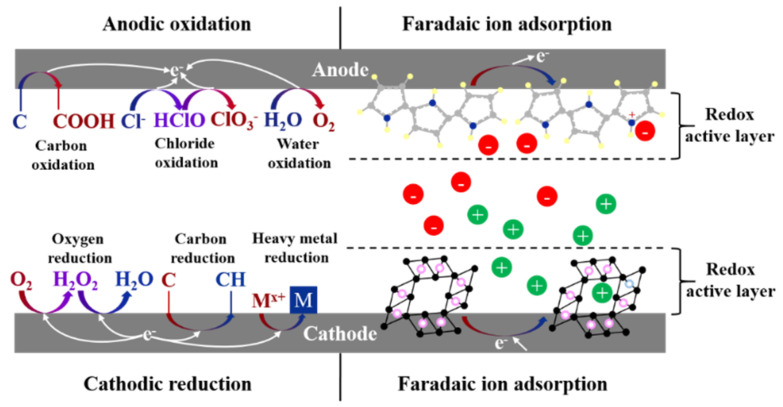
Schematic of Faradaic reactions in CDI, including anodic oxidation, cathodic reduction, and Faradaic ion adsorption via intercalation electrodes.

Redox (Faradaic) reactions may occur inside electrodes with a relatively high working voltage. Three types of Faradaic reactions exist, including anodic oxidation, cathodic reduction, and Faradaic ion adsorption via intercalation electrodes (Figure 12) [454].

Anodic reactions mainly include the oxidation of the carbon electrode, which leads to aldehyde and alcohol group formation on the electrode and ultimately conversion to carbon dioxide; oxidation of chloride to chlorine, hypochlorous acid, and chlorate; and the dissociation of water into oxygen, protons, and hydroxy radicals.

Cathodic reactions mainly include the reduction of oxygen to hydrogen peroxide, and ultimately, water; reduction of heavy metals, if present, to a precipitating form; and the reaction of the carbon electrode with water to form carbon–hydrogen bonds on the electrode surface.

In contrast to the positive effects of reversible Faradaic reactions on intercalation electrodes, the above reactions lead to structure degradation, reduction in long-term stability of the electrode, reduction in energy efficiency, and fluctuations in water quality. Parasitic energy losses (due to irreversible Faradaic reactions) are a significant factor in reducing the efficiency of CDI [455] and should be minimized.

Electrode modification helps suppress irreversible Faradaic reactions. Choi and Choi [456] coated PVDF onto AC powders and bonded them to serve as a CDI electrode. They discovered that elevated PVDF content deactivated the redox functional groups and inhibited Faradaic reactions. A few electrode–polymer composites [457,458] and titania-assisted carbon composites [409,459] have also shown the capability for inhibiting Faradaic reactions. However, the amount of attached titania on the electrode surface should be controlled within a certain range to not reduce capacitance by blocking electrode pores [460]. Furthermore, stability of those titania-functionalized electrodes should be improved to lower the material cost.

Faradaic reactions can also be reduced by adjusting the CDI operational parameters. The optimal applied voltage to avoid trigging Faradaic reactions was found to be 0.8 V in studies under CV mode [372]. Controlling the total applied charge during desalination reduced the Faradaic reactions [461]. CC mode decreased the period of cell operation at a relatively high voltage, thus reducing Faradaic reactions [454]. However, the performance of CC mode depends on the imposed current density. Operation at a low applied current could potentially result in a longer desalination period. Hence, the cell voltage rises slowly, leading to a relatively long operation at high voltage and increased parasitic energy losses [462]. Periodic reversal of polarity also mitigated Faradaic reactions at the expense of incomplete electrode regeneration due to the adsorption of co-ions during regeneration in CDI [454]. By inserting IEM to form MCDI, this co-ion penetration can be largely reduced. The applied voltage during regeneration can be kept low to limit the extra energy consumption.

Alternative CDI architectures help mitigate Faradaic reactions. Attaching CEM onto the cathode helps prevent oxygen from reaching the electrode surface, thus mitigating Faradaic reactions [454]. Removing AEM in MCDI could eliminate oxidative degradation associated with AEM [463]. MCDI with thin IEMs containing high fixed charges could be operated under a relatively low voltage which minimizes the Faradaic reactions without sacrificing the salt removal capacity [464]. In FCDI, pH fluctuation in the flow-electrode channel was negligible if the flow-electrode regeneration was conducted outside the cell, inhibiting the Faradaic reactions [454]. In I-CDI, the functional groups formed in Faradaic reactions served as new adsorption sites and contributed to ion storage capability [454]. Flow-through CDI altered the degree of Faradaic reactions positively toward producing hydrogen peroxide, enabling the potential application of the process for water disinfection and contaminant degradation in bio-wastewater treatment [465].

### 5.11. Status of (M)CDI for Brackish Water Desalination

(M)CDI has been mainly studied at lab-scale, with few efforts reporting large-scale applications and pilot studies [466]. The lab studies suggest, however, that CDI is energy-efficient for the desalination of low-salinity brackish water (salinity below 3000 mg/L) [467] and MCDI even consumes less energy compared to CDI under the same operational conditions [468]. Such results suggest that (M)CDI can be a competitive technology for the desalination of low-salinity brackish water. However, a number of studies demonstrated higher energy consumption for (M)CDI compared to ED and RO when treating brackish water under the same desalination conditions [9,469,470]. A more recent theoretical investigation showed that MCDI can be efficient using the intermittent flow mode with high water recovery [471]. MCDI was able to outperform RO when water recovery was set to 95%, although at this high water recovery, problems from fouling and scaling are more likely.

## 6. Brackish Water Desalination: Which Technology to Choose?

The composition of brackish water varies widely, as do the quality requirements of various applications. The optimal use of brackish water desalination technologies requires definition of both the feed quality and the application quality requirements, and determination of the appropriate niches for each of the technologies. The standards for potable water quality are to some extent location-dependent, with some regions allowing for higher salinities (500–1000 mg/L) and some determining more strict regulations (250 mg/L). Greater flexibility in treatment requirements can be achieved through blending higher-salinity waters with low-salinity waters to meet final water quality goals. The water quality required for industrial applications is heavily dependent upon the process and varies even more. Boilers generally require ultra-pure water, while the water used for hydraulic fracturing in oil and gas development can employ exceedingly high levels of non-precipitating salts as long as there are minimal scale-producing constituents. In desalination for irrigation purposes, both the tolerance of the crops to the salinity and maintaining soil quality (avoiding soil salinization) define desalination requirements [472,473]. The quantity of the treated water required and the availability of concentrate disposal appropriate for those quantities also affect technology selection. Desalination technologies that are efficient for producing potable water for large municipalities may not be economical for small communities and non-centralized treatment. In addition, energy availability and cost and energy efficiency are critically important for large facilities but may be minimally important for small systems such as the desalination of only drinking or cooking waters in a distributed system (e.g., individual home treatment systems). All the technologies reviewed have specific strengths and limitations, and there is no single technique that can be counted as the ultimate solution without consideration of the quantity and quality of both the available waters and the required applications. Table 3 provides a comparison of the main advantages and limitations of the reviewed processes. Thus, to develop the most efficient fit-for-purpose treatment, the desalination process should be selected according to the feed water composition and desalination objectives.

RO is a mature, widely-used technology suitable for the large-scale treatment of brackish water with high salinity (above 5000 mg/L) and seawater. In RO, energy is consumed to transport water from the concentrate to the permeate side, while in ED and (M)CDI, ions are removed from the water to achieve desalinated streams. As a result, the energy efficiency of ED and (M)CDI improves for low-salinity feed waters which require less desalination. At low salinity ranges and high water recovery, ED becomes more energy-efficient than RO [335]. The efficiency of ERDs in RO decreases under such conditions, further reducing the energy-efficiency of RO. Even though RO has dominated the existing desalination processes, the abovementioned limitations suggest that ED, (M)CDI, and NF may have advantages for the treatment of water with lower salinity, desalination for small communities, and selective ion removal.

As noted previously, the lower operational pressure of NF compared to RO makes it a relatively energy-efficient technique for the total desalination of low-salinity waters or the partial treatment of high-salinity waters. The ability to selectively remove multivalent ions suggests that NF is a superior option for the desalination of brackish water with low to moderate salinity that is dominated by such ions [216,217]. For brackish groundwater with moderate salinity (TDS below 6000 mg/L), NF is an effective techno-economical approach to produce potable water with reasonable salinity (800 mg/L) at a higher permeate flux compared to RO [14]. NF is commonly used for water and wastewater treatment, heavy metal removal, arsenic removal from contaminated groundwater, hard brackish water softening, dye and salt removal from industrial wastewater, and partial desalination [474].

ED is the leading electro-membrane technique, with cost-effective operation for the desalination of brackish water with salinity between 1000 and 10,000 mg/L [264]. At water recovery above 80%, the energy requirements of ED is lower than continuous RO and semi-batch RO [335]. Such high water recovery and low SEC make ED a superior option for the desalination of brackish water with moderate salinity (below 3000 mg/L). The low-pressure operation and less required pre-treatment (due to the lower sensitivity of IEMs compared to RO membranes) are among the main advantages of ED over RO. ED is tunable and thus can also be used for partial desalination to reach the required water quality for non-potable water applications. ED can be applied for the selective removal of both monovalent and multivalent ions [475]. Modification of IEMs, electrodes, spacers, cell architecture, unit design, and operational conditions not only have enhanced the performance of ED for brackish water desalination [476,477,478,479] but have also introduced new applications for ED and ED-based processes. A number of such applications include boiler feed water production [480], recovery of valuable metals such as lithium [481,482], selective removal of contaminants [483,484,485], RO concentrate management [486], industrial wastewater treatment [487,488], desalination for agricultural uses [489], zero-liquid discharge desalination [121], ion recovery [490], in-home water treatment with higher water recovery compared to RO [491], acid and base generation [492], nutrient recovery [493,494], and organic acid production [495].

(M)CDI performance has been largely assessed in laboratory studies, but they suggest that the technology can be competitive for the desalination of low-salinity brackish water [496,497]. In addition, (M)CDI typically exhibits a lower capital cost compared to RO [498] and is less susceptible to silica scaling, which can reduce maintenance costs. As noted previously, however, (M)CDI has exhibited higher energy consumption compared to ED and RO when treating brackish water under the same desalination conditions [9,469,470]. Recent studies have shown some potential for improved energy performance at very high water recoveries (95%) [471]. Alternative applications for which (M)CDI may be more efficient include the removal of trace elements from RO permeate compared to second stage RO [499]. In addition, for the desalination of low-salinity feed water (below 1000 ppm) with high water recovery, the thermodynamic energy efficiency (TEE) of RO is relatively low (11–15%), and close to that of (M)CDI with capacitive electrodes [500]. The improvement of process design and electrode material and the implementation of ERDs further reduce energy consumption and make (M)CDI more competitive with RO and ED [349,391,467,501]. (M)CDI with intercalation electrodes can reach a TEE as high as 40% when treating low-salinity water (below 1000 mg/L) [500]. Various cell architectures can be advanced, e.g., FCDI with two pairs of stacked IEMs [502], aiming at cell scaling up to improve desalination capacity and productivity.

Similar to ED, (M)CDI is tunable and selective and thus can be employed for partial desalination. Lab studies indicate that (M)CDI can be used for ultra-pure water production [496], selective removal of scale-forming ions (such as calcium and magnesium ions) for water softening [503], heavy metal removal [496,497], selective removal of nutrients (phosphate and nitrate) [496], water treatment for irrigation [504], water disinfection [497], and the removal of organic compounds through a combination of capacitive and Faradaic adsorption [497] or photocatalytic reactions [505]. FCDI has extensive applications, including water softening [506], ammonia recovery [385,507], nutrient species (phosphate and nitrate) recovery [508,509], heavy metal recovery (copper [510]), lithium extraction [511], divalent and monovalent ion separation [512], and uranium-polluted groundwater treatment [513]. In addition, (M)CDI and ED can be superior alternatives to RO for home-scale potable water desalination, due to the reduced importance of energy efficiency for small production volumes and potentially decreased maintenance costs.

The direct comparison of desalination processes is challenging due to their different levels of readiness and maturity. RO, ED, and NF have been commercialized while (M)CDI is mostly in the research and development stage. With the growing attention of the research community toward the development of novel membranes, electrodes, and operational conditions, it is expected that the application of ED, NF, and (M)CDI will become increasingly competitive for brackish water desalination.

## 7. Conclusions

A variety of technologies are available to provide freshwater from inland brackish waters. The most appropriate technologies are those that can take advantage of the relatively low salinity of brackish waters and can generate water at high recovery rates to minimize concentrate production. Due to generally low energy requirements, membrane desalination processes have been extensively explored for brackish water treatment. Over the past few decades, optimization of the process design and operation, development of novel membranes and electrodes, and the implementation of ERDs have reduced the cost of desalination and made these technologies more feasible. However, membrane performance, fouling and scaling, and concentrate management remain challenges that limit brackish water desalination. In this article, we reviewed the key challenges associated with the membrane processes used for brackish water treatment, and recent research and development efforts to improve those technologies.

Due to the diverse characteristics of brackish water and differences in purification goals, a fit-for-purpose treatment approach considering both the volume and quality of the feed and the requirements of the product water should be considered in selecting the appropriate desalination technique. Technologies that can adapt efficiently to specific purposes and can be tuned to achieve partial desalination for those purposes, such as ED and (M)CDI, have inherent advantages in such applications over the less flexible RO processes, but all processes can be adapted and operated in a manner to achieve appropriate levels of desalination for any desired purpose. The discussion provided insight into the advantages and limitations of these processes that can assist in making appropriate technology choices for any particular desalination application.

## Figures and Tables

**Table 1:** Characteristics of brackish groundwater in the USA [4].

Mean Dissolved Solid, [mg/L]	Dominant Constituents
1800	NaHCO_3_—SO_4_^2−^ accounting for 1/3 of anion concentration
2500	CaSO_4_—Na^+^, Mg^2+^ each accounting for 1/4 of cation concentration
8400	NaCl
1400	Mixture of cations and anions with low solubility—high silica content

**Table 2:** Comparison of various CDI cell architectures.

MCDI Architecture	Main Changes in the Cell Architecture	Characteristic Desalination Mechanisms	Merits	Drawbacks	Recent Advances
Flow-through CDI Figure 11a	Flow direction of feed water is vertical to the charged electrodes.	Both spacer and electrode macropores serve as the flow path. Adsorption occurs only in electrode micropores.	(1) Enabling a more compact cell structure with thinner spacers [379]. (2) Improving desalination rate and salt removal capacity [379].	(1) Major hydraulic pressure loss [380]. (2) Performance degradation from anode oxidation [379].	(1) The enlarged macropores after laser perforations significantly decreased hydraulic pressure loss [380]. (2) Nitrogen purging of the feed water helped remove dissolved oxygen [379].
I-CDI Figure 11b	Positive charges are coated on the surface of cathodes and negative charges are added to the surface of anodes.	Desorption occurs during electrode charging, while adsorption occurs during the regeneration period.	(1) Enhancing electrode stability [351]. (2) Extending cell operation longevity by inhibiting anode oxidation [351].	(1) Relatively low salt removal capacity due to the small working voltage [379].	(1) Amine-treated cathode helped improve salt removal efficiency [353]. (2) Inverted MCDI (I-MCDI) by assembling IEMs into I-CDI possessed higher energy efficiency than MCDI, especially under low working voltage [381].
FCDI Figure 11c	The fixed electrodes are replaced by flowing electrodes (usually activated carbon slurry).	(1) Adsorption occurs in the flow-electrode channel. (2) Both the flow-electrode and the electrolyte in the flow-electrode channel serve as the adsorption sites.	(1) Overcoming the limited desalination capacity [379]. (2) Enabling (semi-)continuous operation [382]. (3) Allowing the desalination of moderate to high concentration brackish water [349].	(1) Poor conductivity in the flow-electrode channel [383].	(1) High flow-electrode content [384], conductive additives such as carbon black [385], carbon nanotubes [386], and plate-type graphite [387], and high flow rate of flow-electrode in flow-electrode channel [16] helped promote cell conductivity.
CDI with intercalation electrodes					
MCDI Architecture	Main changes in the cell architecture	Characteristic desalination mechanisms	Merits	Drawbacks	Recent advances
Desalination battery Figure 11d	Capacitive electrodes are replaced by cation intercalation electrodes (transition metal compounds [388,389], Prussian Blue Analogues (PBAs) [390], and redox-active polymers [73]) and anion intercalation electrodes (Ag/AgCl [389], Bi/BiOCl, and MnO_2_ [391]).	(1) Faradaic adsorption in addition to capacitive adsorption occurs. (2) In CID, one electrode adsorbs cations via positive Faradaic reactions, while the other electrode rejects cations through negative Faradaic reactions. Anions penetrate the AEM and move to the concentrate stream to meet the enriched cations.	(1) Enhancing salt removal capacity [391]. (2) Increasing selectivity towards specific ions [391]. (3) Possessing competitive energy consumption [379].	(1) Reduced desalination performance due to the low electrical conductivity, especially of anion intercalation electrodes [379]. (2) High capital cost [379].	(1) Conductive additives aided intercalation electrode in a CID cell achieved a ten-fold improvement of salt removal rate compared to conventional CID [390]. (2) Long-term cell stability and remarkable adsorption capacity were achieved in an intercalation-anode-assisted I-CDI [392].
HCDI Figure 11e	Capacitive cathode is replaced by cation intercalation electrode, while anode is a capacitive electrode with an AEM.				
CID Figure 11f	Both capacitive electrodes are substituted with cation intercalation electrodes and an AEM is employed as a separator to simultaneously generate desalinated and concentrated streams.				

**Table 3:** Comparison of the desalination processes for brackish water treatment.

Desalination Technique	Advantages	Limitations
RO	Commercialized process High scalability High packing density Applicable for a wide range of feed salinity—most efficient for highly saline brackish water (TDS > 5000 mg/L) Capable of removing both charged and uncharged particles—high quality product water Capable of removing colloidal and organic particles, and some microorganisms Low capital and operational costs at large-scale	High-pressure operation Sensitivity of the membrane to chlorine and high temperature More required pre-treatment Reduced energy efficiency at small-scale and low-salinity feed water Low water recovery at small-scale and low-salinity feed water
NF	Commercialized process High scalability High packing density High water recovery relative to RO Low operational pressure relative to RO Low energy consumption and high permeation relative to RO Energy-efficient for treating moderate-salinity brackish water (TDS < 6000 mg/L) Capable of selective removal of multivalent ions—suitable for water softening Suitable for partial desalination Capable of salt mixture fractionation Low capital and operational costs	High operational pressure relative to ED and MCDI Unable to fully remove monovalent ions Low ion removal efficiency for high-salinity brackish water Post-treatment required
ED	Commercialized process Ease of assembly Low-pressure operation Low sensitivity of the membranes to the feed water quality relative to RO High water recovery especially for low-salinity brackish water (TDS < 3000 mg/L) Energy-efficient for treating low-salinity brackish water (TDS < 3000 mg/L) in small- to medium-scale Tunable—suitable for partial desalination Less required pre-treatment Capable of selective removal of monovalent/multivalent ions Flexible operation—reversal and pulsed electric field modes applicable	Low packing density High cost of ion exchange membranes High costs of electrodes, especially in small-scale Restricted to operation below limiting conditions—increased water dissociation, energy inefficiency, and fouling and scaling once the current exceeds the limiting value Unable to remove contaminants other than charged species—more required post-treatment Relatively high capital costs compared to RO
(M)CDI	Ease of assembly Low-pressure operation Tunable—suitable for partial desalination Capable of selective removal of monovalent/multivalent ions Less susceptible to silica scaling Energy efficient for treating low-salinity brackish water (below 1000 ppm) at high water recovery Flexible cell architectures Capable of energy recovery during regeneration step	Mainly explored in the lab—limited industrial applications Low packing density Poor scalability—most limited to lab-scale and a few pilot-scale applications Relatively high cost of the IEM Unable to remove contaminants other than charged species—more required post-treatment Energy intensive for desalination of high-salinity brackish water at high flow rates Significant parasitic losses from Faradaic reactions under relatively high applied voltage

## Data Availability

Not applicable.
